# DGS-SCSO: Enhancing Sand Cat Swarm Optimization with Dynamic Pinhole Imaging and Golden Sine Algorithm for improved numerical optimization performance

**DOI:** 10.1038/s41598-023-50910-x

**Published:** 2024-01-17

**Authors:** Oluwatayomi Rereloluwa Adegboye, Afi Kekeli Feda, Oluwaseun Racheal Ojekemi, Ephraim Bonah Agyekum, Baseem Khan, Salah Kamel

**Affiliations:** 1 Management Information System Department, University of Mediterranean Karpasia, Mersin-10, Turkey; 2 Management Information System Department, European University of Lefke, Mersin-10, Turkey; 3 Business Administration Department, European University of Lefke, Mersin-10, Turkey; 4 Department of Nuclear and Renewable Energy, Ural Federal University Named After the First President of Russia Boris Yeltsin, 19 Mira Street, Ekaterinburg, Russia 620002; 5 Department of Electrical and Computer Engineering, Hawassa University, Hawassa, Ethiopia; 6 Electrical Engineering Department, Faculty of Engineering, Aswan University, Aswan, 81542 Egypt

**Keywords:** Engineering, Mathematics and computing

## Abstract

This paper introduces DGS-SCSO, a novel optimizer derived from Sand Cat Swarm Optimization (SCSO), aiming to overcome inherent limitations in the original SCSO algorithm. The proposed optimizer integrates Dynamic Pinhole Imaging and Golden Sine Algorithm to mitigate issues like local optima entrapment, premature convergence, and delayed convergence. By leveraging the Dynamic Pinhole Imaging technique, DGS-SCSO enhances the optimizer's global exploration capability, while the Golden Sine Algorithm strategy improves exploitation, facilitating convergence towards optimal solutions. The algorithm's performance is systematically assessed across 20 standard benchmark functions, CEC2019 test functions, and two practical engineering problems. The outcome proves DGS-SCSO's superiority over the original SCSO algorithm, achieving an overall efficiency of 59.66% in 30 dimensions and 76.92% in 50 and 100 dimensions for optimization functions. It also demonstrated competitive results on engineering problems. Statistical analysis, including the Wilcoxon Rank Sum Test and Friedman Test, validate DGS-SCSO efficiency and significant improvement to the compared algorithms.

## Introduction

**Figure 1:**
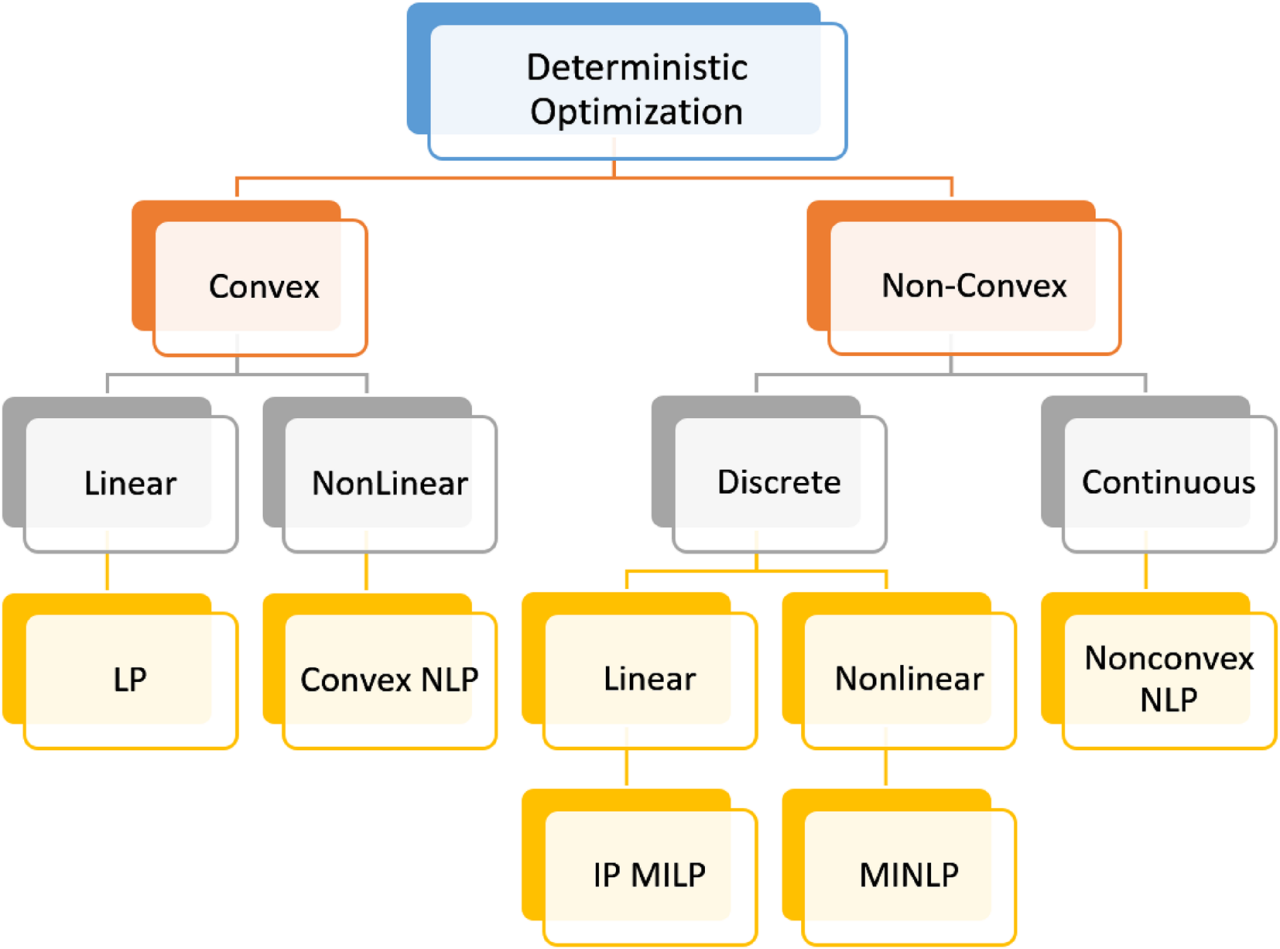
Taxonomy of deterministic optimization algorithms.

Optimization theory is a significant subdivision of computing, which focuses on how to decide the optimum solution from a pool of potential solutions. It offers a structure for defining and resolving complex optimization problems, particularly those with optimization models constrained by significant restrictions, having many objectives, or including complex multivariable systems. Applications of optimization theory can be found in disciplines: computer science^1^, engineering design problem^2–5^,filter design^6–8^, offshore drilling^9^, semi-submersible platform design^10^ and control parameter optimization^11^. Through practice, it has been demonstrated that optimization technologies can increase system effectiveness, appropriately allocate resources, and lower energy usage. A few of the established optimization algorithms are Alpine Skiing Optimization (APS)^12^,Coronavirus Mask Protection Algorithm(CMPA)^13^, and Arithmetic Optimization Algorithm^14^. The superiority of optimization technologies is even more noticeable as the complexity of the optimization problem rises, and precisely, the latter has become significantly more difficult during the last decades. Therefore, the focus of many scholars has shifted to optimization algorithms. Deterministic and meta-heuristic algorithms are the two primary categories into which the new advancements in optimization algorithms fall. Deterministic approaches utilize the problem's analytic characteristics for resolving optimization problems to reach an exact or approximate overall solution^15^. There are various types of deterministic optimization approaches for both convex (with only one optimal solution) and non-convex problems. Techniques for solving convex problems include Linear Programming (LP) and Non-linear Programming (NLP) models. Techniques for solving non-convex problems include Integer Programming (IP), Non-convex Non-linear Programming (NNLP), Mixed-Integer Non-linear Programming (MINLP), and Integer Programming Mixed Integer Linear Programming (IP MILP)^16^. Figure 1 presents the grouping of deterministic optimization algorithms.

Deterministic optimization approaches are effective for a variety of problems, but they might be challenged in providing accurate solutions to problems that are sophisticated, have considerable nonlinearity, or have a significant number of variables^17^. In answer to these limitations, meta-heuristic algorithms (MAs) were introduced. MAs are particularly suitable for complex optimization problems. Since they are non-deterministic, they don't rely on a predetermined set of guidelines or steps to address a particular issue. Instead, they search the solution space and identify the best answers using randomization and probabilistic methods. Due to their adaptability, they can deal with ambiguities in problem formulation and complex challenges. MAs can be grouped into: Swarm Intelligence Algorithms (SI), Evolutionary-Based Algorithms(EB), and Physics-Based Algorithms (PB)^18^. Mathematics or Physics algorithms are created based on mathematical and physical natural principles. One of these is the Gravitational Search Algorithm (GSA)^19^,influenced by Newton's second law and the law of universal gravitation. This algorithm researches the best possible answer for a problem by repeatedly shifting the population's particle position within the search space using their mutual gravitational attraction.The ideal solution is discovered when the particle goes to the ideal spot. EAs are based on the natural biological evolution of species. One example is the Differential Evolution (DE) algorithm introduced by Storn and Price^20^. The DE has drawn a considerable amount of interest and has been applied successfully in a number of contexts. Although using the DE produced better results than using traditional approaches, it showed premature convergence to a local minimum in a complex search space^21^.SI draws its inspiration mostly from biological systems. It mimics the cooperative behavior of sociable animal groups in their attempts to survive. Ant Colony Optimization Algorithm is a popular SI algorithm that mimics how ant colonies behave^22^. The ants’ ability to find direct short routes between their colony and food sources by using chemical pheromone trails as a form of indirect communication is the basis of this behavior. Such an algorithm is typically capable of handling complex problems. Major applications of SI algorithms include the creation of smart strategies for the streamlined transportation of large products and determining the shortest path between two locations^23^and Impulse response filters^24–26^. Another example is the SCSO introduced by Seyyedabbasi and Kiani, which mimics the lifestyle and unique abilities of the sand cat^27^. Although SCSO has been employed to address engineering optimization challenges and several test functions, there is still the problem of being stuck in the local optimum, premature convergence, and delayed convergence because of sound frequency guiding each sand cat toward the prey. Based on the frequency intensity, the sand cat can either search or attack the prey; this serves as a prey-following mechanism that may trap the sand cat in local solution, causing poor convergence.

In response, this study proposes Sand Cat Swarm Optimization based on Dynamic Pinhole Imaging (DPI) and Golden Sine Algorithm (Gold-SA) called DGS-SCSO. While there is a plethora of existing metaheuristic algorithms and improved versions of this algorithm, the novelty of the DGS-SCSO algorithm lies in its unique combination of Dynamic Pinhole Imaging (DPI) and the Golden Sine Algorithm (Gold-SA) with the existing Sand Cat Swarm Optimization (SCSO) which is not found in previously modified versions of SCSO. Furthermore, the “No Free Lunch theorem states that no single algorithm can be suitable for every problem^28^”; hence, DGS-SCSO strategically utilizes DPI, a more precise version of opposition-based learning, to initialize a population with diverse solutions, improving the chances of locating the global optimal. Meanwhile, Gold-SA is used to change the location of the best sand cat to get closer to the optimal solution and encourage rapid convergence and exploitation. Gold-SA facilitates a continual decrease in the problem space, allowing the algorithm to concentrate on areas more likely to yield globally optimal solutions.The integration of DPI and Gold-SA into SCSO not only distinguishes DGS-SCSO from existing algorithms but also addresses the algorithmic deficiencies observed in the original SCSO. Making DGS-SCSO a valuable addition to the existing metaheuristic algorithms in the literature. This research makes several significant contributions, including the proposal of a new optimization algorithm named DGS-SCSO. The effectiveness of DGS-SCSO is assessed on 20 classic benchmark functions, 10 CEC2019 competition benchmark functions, and two engineering problems. To assess the effectiveness of DGS-SCSO, the algorithm is contrasted against seven recent metaheuristic algorithms. The results of DGS-SCSO are analysed and interpreted using several methods, ensuring a detailed assessment of the new optimizer's effectiveness.

The paper is thus structured: Sect. “Related work” provides an overview of relevant research, while the original SCSO algorithm is presented in Sects. “Original SCSO”. “Proposed DGS-SCSO” explains the improvement strategies and introduces the proposed DGS-SCSO algorithm. Sections “Analysis of complexity”, “Experiments and discussion” and “Application of engineering problem” describe the complexity analysis, experiments, and conclusions, respectively.

## Related work

Despite the fact that the method was introduced recently, some studies have been done on improving SCSO and tackling the previously mentioned limitations. Arasteh et al. introduced a novel variant of the SCSO algorithm for software module clustering^29^. Their goal was to provide optimal clusters for source codes' dependency graphs. SCSO was revised to maximize its position-updating stage to obtain better results. Another major change was to add a controlled mutation technique, such as that seen in the Genetic algorithm (GA), to boost heterogeneity and efficiency. Ten common functions were used to rate how well the suggested method performed. In terms of overall success, convergence time, and modularization quality, the proposed algorithm outperformed the other algorithms compared to it. Li et al. introduced a Stochastic and Elite based SCSO (SE-SCSO)^30^. In the proposed SE-SCSO, Li et al. improved the convergence speed, local exploitation, and exploration of the traditional SCSO with a periodic non-linear adjustment process. Overall efficiency and capacity of convergence were enhanced by using the opposition and reflection learning processes. The validity of the suggested improvements was supported by the experimental results. Iraji et al. suggested a hybridized strategy based on chaotic SCSO and pattern search named CSCPS for their study^31^. The chaotic sequence was used to increase the SCSO approach’s exploring capability while also preventing untimely convergence. Mathematical test functions were used to assess the efficiency of the new CSCPS optimizer. CSCPS had overall better performance. Wu et al. introduced a modified version of SCSO (MSCSO). In the MSCSO algorithm, sand cats’ position updating is done by wandering techniques^32^. The triangular walking (TW) technique for searching and The Levy flight walking (LFW) technique to attack prey. Sand cats employ a Roulette Wheel selection algorithm to calculate their distance from their prey in order to determine the best trajectory before updating their position in accordance with the trigonometric function calculation theory. The MSCSO was evaluated using the CEC2014 functions and 23 additional functions, and it showed superior exploration capacity. The technical applicability of the suggested strategy was finally proven by applying it successfully to test seven engineering problems. Jovanovic et al. suggested a novel SCSO technique to improve the efficacy of the extreme learning machine (ELM) classifier^33^. The concept of “exhausted solutions” derived from the popular Artificial Bee Colony Algorithm is included in the algorithm. The suggested approach has been verified on two distinguished datasets, and the improvements in performance are shown by contrasting the outcomes with those of other optimizers that operate in a comparable manner. Lu et al. developed an Improved SCSO (ISCSO)^34^. They employed logistic mapping to initialize the population and obtained a population more equally distributed, which enhanced the algorithm convergence and optimization precision. In order to solve the SCSO algorithm's constraint and poor accuracy when addressing complex multivariate functions with numerous peaks, a water wave dynamic evolution component was incorporated. The utilization of water wave dynamics lessened the blindness of individuals who are trailing one another. Finally, the weighted adaptive algorithm was taken into consideration to smoothen the switch between global search and local exploitation. The ISCSO performed better overall when compared to other traditional algorithms in tests, and it required a few iterations to converge to a comparable precision.

## Original SCSO

The SCSO, introduced in 2022 by Seyyedabbasi and Kiani, is a MA that takes inspiration from the hunting patterns and biological characteristics of sand cats^27^. These felines require 10% more food than domestic cats and have developed unique hunting mechanisms to satisfy their needs. With their exceptional hearing capabilities, they can perceive low-frequency sounds and detect prey movements underground. Additionally, they possess remarkable endurance, allowing them to cover long distances without rest. Drawing from these traits, SCSO imitates the two distinct phases of sand cats' hunting process: foraging and catching the prey. SCSO portrays the problem variables as represented by the attributes of sand cats, which are structured as vectors. In the problem space, a single sand cat is modeledas a 1 × ${\text{dim}}$ array that encodes the search space, where ${\text{dim}}$ denotes dimension. Notably, each variable value $({x}_{1, }{x}_{2 }, \dots ,{x}_{{\text{dim}})}$ is denoted by a floating-point number that falls within the specified lower and upper bounds. To initialize the SCSO algorithm, a candidate matrix is constructed by assembling a population of sand cats with a size of $N*dim$ where $N$ denotes population of cats, in proportion to the dimensions of the problem.

Furthermore, the SCSO algorithm assesses the fitness cost of every sand cat using a designated fitness function that corresponds to the problem characteristics. The optimization process aims to identify the optimal values of the parameters (variables) via this function, which returns a corresponding solution for every single sand cat. Finally, the sand cat having the best fitness cost up to that point is selected as the best solution and the remaining sand cats adjust their positions accordingly in the following iteration. This mechanism imitates the behavior of sand cats, who tend to follow the most successful hunter in their group. Notably, the SCSO algorithm avoids excessive memory usage by only storing the best solution of every iteration, which can be thought of as the sand cat nearest to the prey. This iterative process is repeated until the desired level of optimization is achieved. The search technique of the SCSO algorithm was modeled after the low-frequency noise emission-based on the hunting method used by sand cats. The algorithm exhibits a single sand cat's solution as ${X}_{i }= ({x}_{i1, }{x}_{i2 }, \dots ,{x}_{{\text{idim}})}$, and leverages sand cats' low-frequency hearing ability to set every cat's sensitivity range. To aid the cats in approaching their goal without losing or passing it, the value of the sensitivity range (abbreviated as $\overrightarrow{{r}_{G}}$) linearly declines from 2 to 0 kHz as the iterations go on (According to Eq. 1). The ${S}_{M}$ number, which represents the hearing qualities of sand cats, is initially set to 2, but it can be adapted to the problem being solved to decide how quickly the agents will act. This demonstrates the algorithm's adaptability and flexibility. The vector $\overrightarrow{{\text{R}}}$, derived in Eq. (2), is another important parameter for regulating the switch from exploration to exploitation. The adaptive approach optimizes the algorithm's performance by ensuring a smooth transition between the two stages.

1 $$\overrightarrow{{r}_{G}}={s}_{M}-\left(\frac{2\times {S}_{M}\times {\text{ it }}_{{\text{c}}}}{{\text{ it }}_{\text{Max }}+{\text{ it }}_{max}}\right)$$

2 $$\overrightarrow{{\text{R}}}=2 \times \overrightarrow{{r}_{G}}\times rand\,(\mathrm{0,1})-\overrightarrow{{r}_{G}}$$

where ${\text{it }}_{{\text{c}}}$ and ${\text{it }}_{\text{Max}}$ denotes respectively the current and the maximum iterations. The search space is initialized at random within the specified borders. A unique sensitivity range ($\overrightarrow{{\text{r}}}$ ) is assigned to every sand cat in order to escape the local optimum, as seen in Eq. (3).

3 $$\overrightarrow{{\text{r}}}=\overrightarrow{{r}_{G}}\times rand\,(\mathrm{0,1})$$

The positions of each sand cat are upgraded based on its present position ($\overrightarrow{{{\text{P}}}_{c}}$), sensitivity range ($\overrightarrow{{\text{r}}}$) and best candidate position $\overrightarrow{{({\text{P}}}_{bc})}$ as shown in Eq. (4).

4 $$\overrightarrow{{\text{P}}}\left(t+1\right)=\overrightarrow{{\text{r}}} \times ( \overrightarrow{{{\text{P}}}_{bc}}\left(t\right)-rand\,\left(\mathrm{0,1}\right)\times \overrightarrow{{{\text{P}}}_{c}}\left(t\right))$$

The distance ($\overrightarrow{{{\text{P}}}_{rnd}}$ ) from the current location $\overrightarrow{{{\text{P}}}_{c}}$ to the best candidate position $\overrightarrow{{{\text{P}}}_{bc}}$ of each sand cat is determined by applying Eq. (5). An arbitrary angle $\alpha$, is chosen using the Roulette Wheel selection algorithm to determine the trajectory’s orientation. The random angle ranges from 0° to 360° and has a value between -1 and 1. This allows every individual to move across the search space in a distinct circular pattern. $\alpha$ is utilized to change the position of each sand cat, as indicated in Eq. (5), which guides them toward the prey. The prey is then caught with Eq. (6).

5 $$\overrightarrow{{{\text{P}}}_{rnd}}=\left|rand\,\left(\mathrm{0,1}\right)\times \overrightarrow{{{\text{P}}}_{bc}}\left(t\right)- \overrightarrow{{{\text{P}}}_{c}}\left(t\right)\right|$$

6 $$\overrightarrow{\mathrm{P }}\left(t+1\right)=\overrightarrow{{{\text{P}}}_{bc}}\left(t\right)- \overrightarrow{{\text{r}}} \times \overrightarrow{{{\text{P}}}_{rnd }} \times {\text{cos}}(\alpha )$$

The SCSO algorithm uses adaptive parameters $\overrightarrow{{r}_{G}}$ and R to monitor the tradeoff necessary between the local and global search. To achieve this,$\overrightarrow{{r}_{G}}$ linearly and progressively declines from 2 to 0 as with iterations. Meanwhile, the parameter R is generated arbitrarily from the interval [− 4, 4]. If $|R|$ is inferior or equal to 1 the individual cat can catch the prey; if not, search continues as given Eq. (7).

7 $$\overrightarrow{\mathrm{P }}\left(t+1\right)=\left\{\begin{array}{cc}\overrightarrow{{P}_{{\text{b}}}}(t)-\overrightarrow{{{\text{P}}}_{rnd}}\times {\text{cos}}(\alpha )\times \overrightarrow{r}& |R|\le 1;\mathrm{ exploitation }\\ \overrightarrow{r}\cdot \left(\overrightarrow{{{\text{P}}}_{bc}}(t)-{\text{rand\,}}(\mathrm{0,1})\times \overrightarrow{{P}_{c}}({\text{t}})\right)& |R|>1;\mathrm{ exploration}\end{array}\right.$$

## Proposed DGS-SCSO

### Dynamic Pinhole Imaging strategy (DPI)

**Figure 2:**
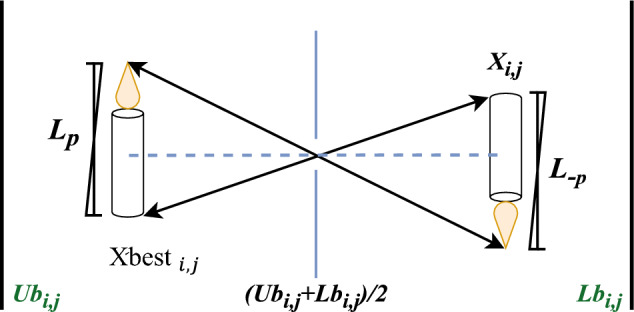
Dynamic pinhole imaging strategy.

The overall performance of population-based metaheuristic algorithms is significantly influenced by their initialization phas^35^. A poor initialization may cause the algorithm to explore unpromising regions, subjecting it to the local solution. On the other hand, efficient population initialization can considerably increase precision and algorithm convergence speed. When the starting collection of solutions is located close to the best solution, there is a greater chance of locating the global optimum with a smaller search effort. Opposition-based Learning (OBL) is a technique that draws its inspiration from the opposite relationship between real-world entities^36,37^. The concept was first introduced in 2005, and it has piqued significant research interest. OBL has been successfully applied to enhance algorithms’ population initialization. The fundamental idea behind OBL is to jointly explore an arbitrary direction and its mirror image while seeking an unknown global optimum. The likelihood of two individuals being closer to the optimal solution is 50% if they are positioned at the opposite location of each other. As a result, only a few operations are needed to create a population of greater quality. This technique is analogous to the pinhole imaging theory in optics. Pinhole imaging is more precise than standard Opposite-based learning and can generate a wider range of opposing points^38^. A theoretical representation of pinhole imaging is shown in Fig. 2. The following equation is obtained by applying the model in Fig. 2 to the population's search space Eq. (8):

8 $$\frac{{\text{Xbest }}_{i,j}-\left(U{b}_{i,j}+L{b}_{i,j}\right)/2}{\left(U{b}_{i,j}+L{b}_{i,j}\right)/2-{X}_{i,j}}=\frac{{L}_{p}}{{L}_{-p}}$$

where the location of the best search agent is denoted as ${\text{Xbest }}_{i,j}$, while the opposite point is represented by ${X}_{i,j}$. The i-th agent in the j-th dimension has lower and upper bounds denoted as $L{b}_{i,j}$ and $U{b}_{i,j}$ respectively. Furthermore, ${L}_{p}$ stands for the size of the candle at the best location and ${L}_{-p}$ the size of the one at the opposite location. Although the candle’s location matches that of the search agent, the search agent’s point has no efficient length. As a result, $K$ can be assigned as a variable to represent the two candles' ratio, as shown in Eq. (9).

9 $${X}_{i,j}=\frac{(K+1)\left(U{b}_{i,j}+L{b}_{i,j}\right)-2{\text{Xbest }}_{i,j}}{2K}$$

By analyzing Eq. (9), it is apparent that when both candles have equal lengths, the strategy becomes a simple reverse learning approach. Modifying effectively the value of $K$ can alter the location of the opposing point, which leads to greater search opportunities for the individuals.

### Golden Sine algorithm (Gold-SA)

The Gold-SA is based on the “sine function in mathematics,” and it also utilizes the golden ratio to seek a superior answer in the problem space. The sine function's range is within − 1 to 1, and it has a period of 2π. As ${x}_{1}$ changes, its associated variable ${y}_{1}$ also does. Through the golden ratio, the problem domain can be continually decreased, and the algorithm can focus on areas where the likelihood of producing the globally acceptable answer is higher, resulting in faster convergence Eq. (10).

10 $${X}_{i,j}(t+1)={X}_{i,j}(t)\times \left|{\text{sin}}\left({p}_{1}\right)\right|-{p}_{2}\times {\text{sin}}\left({p}_{1}\right)\times \left|{d}_{1}\times {X}_{{\text{best}},j}(t)-{d}_{2}\times {X}_{id}(t)\right|$$

The formula involves two arbitrary values ${p}_{1}\in$[0, 2π], and ${p}_{2}\in$[0, π], ${X}_{i,j}$ denote the current individual, ${X}_{{\text{best}},j}$ denoted the best individual and two coefficient factors ${d}_{1}$ and ${d}_{2}$ that is determined by Eqs. (11) and (12)

11 $${d}_{1}=a\times \tau +b\times \left(1-\tau \right)$$

12 $${d}_{2}=a\times (1-\tau )+b\times \tau $$

where $a$ and $b$ are initialized respectively to -π and π. The golden ratio, τ is $(\surd {\text{p}}5 - 1)/2$.

### Implementation of proposed DGS-SCSO

**Figure 3:**
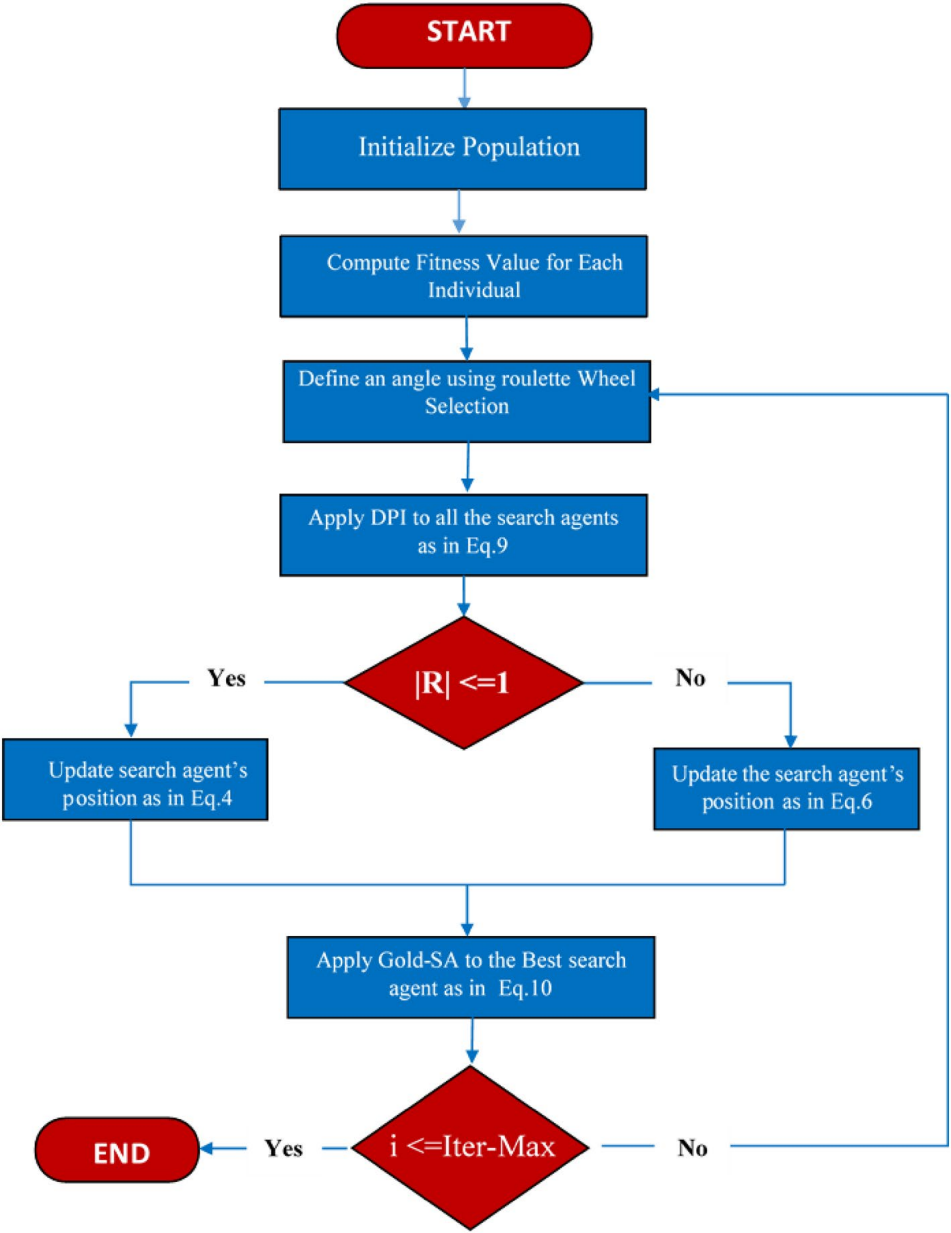
Flowchart of DGS-SCSO.

The original SCSO algorithm appears to have a tendency to converge too quickly to local optima, which can limit its capacity to local the global optimal. Additionally, the algorithm's convergence speed may be slow, which could also hinder its effectiveness. To address these issues, two modifications have been proposed: DPI and Gold-SA. DPI is intended to expand the optimizers' global capacity to escape the trap of the local optimal. Gold-SA, on the other hand, is designed to enhance the algorithm's local search ability, enabling it to quickly find optimal solutions in the search area. By incorporating these modifications into the original SCSO algorithm, it is expected that the algorithm's performance will be significantly improved. Specifically, the modifications should help to strike the best transition from exploration to exploitation, thereby increasing the population’s diversity and making it more likely that the algorithm will converge to global optima. The pseudo-code for DGS-SCSO is provided in algorithm 1. The flow chart of DGS-SCSO is given in Fig. 3.

**Algorithm 1:**
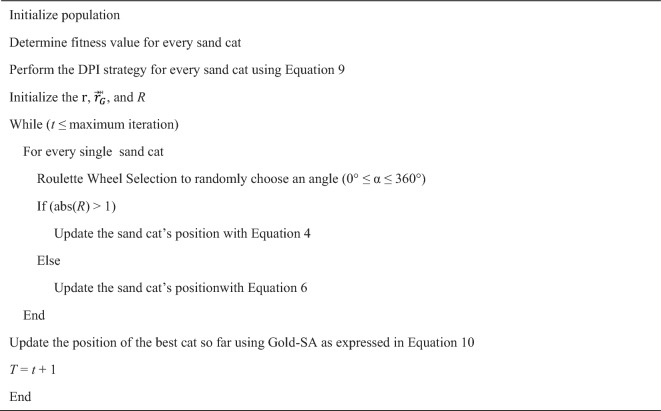
DGS-SCSO Algorithm Pseudo-Code.

## Analysis of complexity

The initialization phase has a computing cost of ${\text{O}}(N\times D)$, where $N$ denotes the population size, and $D$ is the dimension size. During this phase, SCSO generates the sand cats at random throughout the problem space. Following that, DGS-SCSO assesses each individual's fitness over the course of the entire iteration with a complexity of ${\text{O}}(T\times N\times D)$, with $T$ denoting the number of iterations. Finally, to reach the best option, we employed Gold-SA and DPI. Therefore, these phases' computational complexity is ${\text{O}}(3\times T\times N\times D)$. In conclusion, the DGS-SCSO's overall computational complexity is ${\text{O}}(T\times N\times D)$.

## Experiments and discussion

We assess the effectiveness of the suggested DGS-SCSO method by subjecting it to 20 commonly used benchmark functions and the 10-functions CEC 2019 competition test suite. Additionally, the effectiveness of the method is assessed by using it to solve two engineering problems. The experimental setup and benchmark function properties are elucidated in detail in the following section, followed by a comprehensive analysis and commentary on the statistical findings of the 30 benchmark functions. Finally, the benefits of utilizing DGS-SCSO are demonstrated through its application to the aforementioned engineering design problems.

### Function definition

The study employed a total of 30 test functions, including 10 CEC 2019 test functions and 20 widely used benchmark functions. Based on their properties, the 20 classical functions were separated into three categories. The functions F1 through F7 are useful for assessing the exploitability of algorithms because they are unimodal, they possesses a single global optimum, and lack any local optima. The functions F8 through F13 were beneficial for assessing algorithms' exploration and local minimum avoidance capabilities. The fixed multipeaked functions F14 through F20 have different low-dimensional local optima, and they are used to assess the stability and algorithms’ capability of avoiding local optimum.

The study employed 10 functions (F21–F30) from the CEC 2019 benchmark suite in addition to the traditional functions. These functions have been shifted and rotated, adding complexity above the conventional functions. The specifics of each function are supplied in Tables 1 and 2, and the optimal fitness of each function is marked by f_min_. The primary goal of this section is to assess the DGS-SCSO algorithm's search capability on a variety of complicated functions with various properties.

**Table 1:** Specifics of the 20 Classic Functions.

Function	Dimension	Range	Fmin
${f}_{1}(x)={\sum }_{i=1}^{n} {x}_{i}^{2}$	30	$[-\mathrm{100,100}]$	0
${f}_{2}(x)={\sum }_{{\text{i}}=1}^{n} \left|{x}_{i}\right|+{\prod }_{i=1}^{n} \left|{x}_{i}\right|$	30	$[-\mathrm{10,10}]$	0
${f}_{3}(x)={\sum }_{i=1}^{n} {\left({\sum }_{j-1}^{i} {x}_{j}\right)}^{2}$	30	$[-\mathrm{100,100}]$	0
${f}_{4}(x)={{\text{min}}}_{i} \left\{\left|{x}_{i}\right|,1\le i\le n\right\}$	30	$[-\mathrm{100,100}]$	0
${f}_{5}(x)=\sum_{i=1}^{d} \sum_{j=1}^{i} {x}_{j}^{2}$	30	$[-\mathrm{65.536,65.536}]$	0
${f}_{6}(x)={\sum }_{i=1}^{n} {\left(\left[{x}_{i}+0.5\right]\right)}^{2}$	30	$[-\mathrm{100,100}]$	0
${f}_{7}(x)={\sum }_{i=1}^{n} i{x}_{i}^{4}+{\text{random}}[\mathrm{0,1})$	30	$[-\mathrm{1.28,1.28}]$	0
${f}_{8}(x)=1-{\text{cos}}\left(2\pi \sqrt{\sum_{i=1}^{d} {x}_{i}^{2}}\right)+0.1\sqrt{\sum_{i=1}^{d} {x}_{i}^{2}}$	30	$[-\mathrm{100,100}]$	0
${f}_{9}(x)={\sum }_{i=1}^{n} \left[{x}_{i}^{2}-10{\text{cos}}\left(2\pi {x}_{i}\right)+10\right]$	30	$[-\mathrm{5.12,5.12}]$	0
${f}_{10}(x)=-20{\text{exp}}\left(-0.2\sqrt{\frac{1}{n}{\sum }_{i=1}^{n} {x}_{i}^{2}}\right)-{\text{exp}}\left((1/n){\sum }_{i=1}^{n} {\text{cos}}\left(2\pi {x}_{i}\right)\right)+20+e$	30	$[-\mathrm{32,32}]$	0
${f}_{11}(x)=1/4000{\sum }_{i=1}^{n} \sum {x}_{i}^{2}-{\prod }_{i=1}^{n} {\text{cos}}\left({x}_{i}/\sqrt{i}\right)+1$	30	$[-\mathrm{600,600}]$	0
${f}_{12}(x)=\pi /n\left\{{\sum }_{i=1}^{n-1} {\left({y}_{i}-1\right)}^{2}\left[1+10{{\text{sin}}}^{2}\left(\pi {y}_{i+1}\right)\right]+{\left({y}_{n}-1\right)}^{2}\right\}$	30	$[-\mathrm{50,50}]$	0
$$+{\sum }_{i=1}^{n} u\left({x}_{i},\mathrm{10,100,4}\right)+\pi /n10{\text{sin}}\left(\pi {y}_{1}\right)$$			
$${y}_{i}=1+{x}_{i}+(1/4)u\left({x}_{i},a,k,m\right)=\left\{\begin{array}{ll}k{\left({x}_{i}-a\right)}^{m}& {x}_{i}>a\\ 0& -a<{x}_{i}<a\\ k{\left(-{x}_{i}-a\right)}^{m}& {x}_{i}<-a\end{array}\right.$$			
${f}_{13}(x)=0.1\left\{{\sum }_{i=1}^{n} {\left({x}_{i}-1\right)}^{2}\left[1+{{\text{sin}}}^{2}\left(3\pi {x}_{i}+1\right)\right]+{\left({x}_{n}-1\right)}^{2}\left[1+{{\text{sin}}}^{2}\left(2\pi {x}_{n}\right)\right]\right\}+0.1{{\text{sin}}}^{2}\left(3\pi {x}_{1}\right)+{\sum }_{i=1}^{n} u\left({x}_{i},\mathrm{5,100,4}\right)$	30	$[-\mathrm{50,50}]$	0
${f}_{14}(x)=\left|{x}^{2}+{y}^{2}+xy\right|+|{\text{sin}}(x)|+|{\text{cos}}(y)|$	2	$[-\mathrm{500,500}]$	1
${f}_{15}(x)={{\text{sin}}}^{2}(3\pi x)+(x-1{)}^{2}\left(1+{{\text{sin}}}^{2}(3\pi y)\right)+(y-1{)}^{2}\left(1+{{\text{sin}}}^{2}(2\pi y)\right)$	4	$[-\mathrm{10,10}]$	0
${f}_{16}(x)=4{x}_{1}^{2}-2.1{x}_{1}^{4}+1/3{x}_{1}^{6}+{x}_{1}{x}_{2}-4{x}_{2}^{2}+4{x}_{2}^{4}$	2	$[-\mathrm{5,5}]$	− 1.0316
${f}_{17}(x)={\left({x}_{2}-5.1/4{\pi }^{2}{x}_{1}^{2}+5/\pi {x}_{1}-6\right)}^{2}+10(1-(1/8\pi )){\text{cos}}{x}_{1}+10$	2	$[-\mathrm{5,5}]$	0.398
${f}_{18}(x)=\left[1+{\left({x}_{1}+{x}_{2}+1\right)}^{2}\left(19-14{x}_{1}+3{x}_{1}^{2}-14{x}_{2}+6{x}_{1}{x}_{2}+3{x}_{2}^{2}\right)\right]\times$	2	$[-\mathrm{2,2}]$	3
$$\left[30+{\left(2{x}_{1}-3{x}_{2}\right)}^{2}\times \left(18-32{x}_{1}+12{x}_{1}^{2}+48{x}_{2}-36{x}_{1}{x}_{2}+27{x}_{2}^{2}\right)\right]$$			
${f}_{19}(x)=-{\sum }_{i=1}^{4} {c}_{i}{\text{exp}}\left[-{\sum }_{j=1}^{3} {a}_{ij}{\left({x}_{j}-{p}_{ij}\right)}^{2}\right]$	3	$[\mathrm{1,3}]$	− 3.86
${f}_{20}(x)={x}^{2}+2{y}^{2}-0.3{\text{cos}}(3\pi x){\text{cos}}(4\pi y)+0.3$	2	$[-\mathrm{100,100}]$	0

**Table 2:** Specifics of the 10 CEC 2019 functions.

No	Function names	$Fmin$	Dim	Range
$$\begin{array}{l}{f}_{21}\\ \end{array}$$	Storn's Chebyshev polynomial fitting problem	1	9	[$-\mathrm{8,192},\mathrm{ 8,192}]$
$$\begin{array}{l}{f}_{22}\\ \end{array}$$	Inverse Hilbert matrix problem	1	16	[$-\mathrm{16,384},\mathrm{ 16,384}]$
$$\begin{array}{l}{f}_{23}\\ \end{array}$$	Lennard-Jones minimum energy cluster	1	18	$[-\mathrm{4,4}]$
$$\begin{array}{l}{f}_{24}\\ \end{array}$$	Rastrigin's function	1	10	$[-\mathrm{100,100}]$
$$\begin{array}{l}{f}_{25}\\ \end{array}$$	Griewank's function	1	10	$[-\mathrm{100,100}]$
$$\begin{array}{l}{f}_{26}\\ \end{array}$$	Weierstrass function	1	10	$[-\mathrm{100,100}]$
$$\begin{array}{l}{f}_{27}\\ \end{array}$$	Modified Schwefel’s function	1	10	$[-\mathrm{100,100}]$
$$\begin{array}{l}{f}_{28}\\ \end{array}$$	Expanded Schaffer’s F6 function	1	10	$[-\mathrm{100,100}]$
$$\begin{array}{l}{f}_{29}\\ \end{array}$$	Happy Cat function	1	10	$[-\mathrm{100,100}]$
$$\begin{array}{l}{f}_{30}\\ \end{array}$$	Ackley function	1	10	$[-\mathrm{100,100}]$

### Experimental setup

Thirty different test functions where used to assess how well the DGS-SCSO optimization algorithm performed. To confirm the accuracy of the outcomes, the proposed algorithm was contrasted against several other algorithms, including SCSO^27^, Artificial Electric Field Algorithm (AEFA)^39^, Honey Badger Algorithm (HBA)^40^, Hybrid Butterfly Optimization Algorithm with Particle Swarm Optimization (HPSOBOA)^41^, Quadratic interpolation Salp Swarm-Based local escape operator (QSSALEO)^42^, Time-Based Leadership Salp-Based Algorithm with Competitive Learning (TBLSBCL)^43^, Transient Search Algorithm (TSO)^44^. We established the maximum iteration to be 1000, the population size to be 30, and the dimension size to be as mentioned in Tables 1 and 2. Additionally, we conducted 30 independent runs for the experimental setup. The best results are indicated in bold. Table 3 presents the specific parameter settings for the algorithms used in the experiment.

**Table 3:** Parameter settings.

Algorithms	Parameter setting
DGS-SCSO	Roulette wheel selection [0, 360], $C=0.35,SM=2, K$= 1.5 × 10^4^
SCSO^27^	Roulette wheel selection [0, 360],$C=0.35,SM=2$
AEFA ^39^	Coulombs constant ${k}_{0}=500$,$\gamma =30$
HBA^40^	$\beta =6$, $C=2$
HPSOBOA^41^	$$\begin{array}{c}{a}_{\text{first }}=0.1,{a}_{\text{final }}=0.3,c(0)=0.01,p=0.6\\ x(0)=0.315,\rho =0.295,{c}_{1}={c}_{2}=0.5\end{array}$$
QSSALEO^42^	Light absorption coefficient $\zeta =1$, step size $s=0.2$
TBLSBCL^43^	$\phi =0.3$, $c1=$ [2/e,2]
TSO^44^	$k=2, z\in [\mathrm{0,2}]$

### Statistical result analysis

The DGS-SCSO algorithm exhibits noteworthy results when compared to other metaheuristic algorithms across Tables 4, 5, and 6. In Table 4, the dimension of the function remained as detailed in Tables 1 and 2; in Tables 5 and 6, dimensions are set to 50 and 100, which increases the complexity of the test suite function. In Table 4, DGS-SCSO achieves superior average values (AVG) and remarkable stability with smaller standard deviation (STD) on various functions, indicating consistent and robust performance. In F1, F3, and F5, from the unimodal function, DGS-SCSO obtained the theoretically optimal solution. This is in contrast to SCSO, QSSALEO, and TSO, which obtained the near-ideal solution. In F2, F4, and F7, DGS-SCSO outperformed HBA and TBLSBCL, obtaining the best solution. For the multimodal functions, DGS-SCSO is shown to outperform AEFA, HBA, and TSO for F8, F9, and F11, indicating its superior ability to handle complex and challenging optimization problems with multiple local optima. Additionally, it performs better than SCSO for F15, F17, and F19. The results for the CEC 2019 functions show that DGS-SCSO produces better results than the compared optimizers in six of the functions (F23, F24, F25, F26, F28, and F29), suggesting its effectiveness in handling a diverse set of optimization problems.

**Table 4:** Result of different algorithms on 30 functions.

		DGS-SCSO	SCSO	AEFA	HBA	HPSOBOA	QSSALEO	TBLSBCL	TSO
F1	AVG	**0**	2.64E-143	4.77E-21	4.03E-70	8.39E-9	5.49E-14	9.87E-9	4.29E-153
	STD	**0**	4.89E-144	3.45E-21	8.97E-71	3.61E-9	2.02E-14	1.73E-9	7.96E-154
F2	AVG	**1.82E-217**	5.01E-73	1.04E + 2	9.88E-40	4.24E-1	9.13E-8	1.41	9.57E-63
	STD	**0**	9.31E-74	4.57E + 1	2.62E-40	3.21E-1	6.42E-8	1.35	5.15E-62
F3	AVG	**0**	2.51E-140	1.75E + 3	5.92E-33	4.48E-7	1.62E-14	7.73E + 1	2.88E-139
	STD	**0**	4.67E-141	4.43E + 2	1.20E-33	1.96E-7	1.59E-14	5.35E + 1	7.44E-140
F4	AVG	**1.61E-241**	1.41E-70	1.48	1.31E-23	1.21E-5	1.43E-7	6.84	2.52E-72
	STD	**0**	2.63E-71	1.00	2.44E-24	8.52E-6	9.12E-8	2.43	4.68E-73
F5	AVG	**0**	4.96E-150	2.18	3.67E-65	2.55E-7	2.40E-13	4.50	3.52E-114
	STD	**0**	9.21E-151	**0**	6.82E-66	7.00E-7	9.79E-14	1.04	6.53E-115
F6	AVG	6.15	6.32	**2.39E-21**	9.38E-5	6.28	1.28E-8	9.67E-9	9.16E-5
	STD	5.12E-1	3.11E-1	**1.87E-21**	3.89E-6	4.80E-1	2.96E-9	2.08E-9	4.30E-5
F7	AVG	**2.69E-5**	4.20E-5	1.63	2.79E-3	3.76E-4	1.11E-4	6.96E-2	5.76E-5
	STD	**2.50E-5**	3.67E-5	5.29E-1	2.45E-3	3.13E-4	1.11E-4	2.22E-2	4.31E-5
F8	AVG	**0**	1.70E-78	1.39	1.13E-1	4.24E-6	2.11E-8	8.67E-1	3.03E-78
	STD	**0**	3.54E-79	3.05E-1	7.27E-2	4.06E-6	1.77E-8	1.47E-1	7.54E-79
F9	AVG	**0**	**0**	4.15E + 1	1.54	2.33E-6	5.43E-14	4.08E + 1	**0**
	STD	**0**	**0**	1.29E + 1	1.38E + 1	5.66E-6	1.33E-14	1.10E + 1	**0**
F10	AVG	**4.44E-16**	**4.44E-16**	1.45E-1	4.77	3.74E-5	5.93E-8	2.92	**4.44E-16**
	STD	**9.86E-32**	**9.86E-32**	3.76E-1	7.31	3.15E-5	3.96E-8	5.91E-1	**9.86E-32**
F11	AVG	**0**	**0**	3.93E-1	2.46E-5	3.20E-10	2.34E-14	1.36E-2	**0**
	STD	**0**	**0**	5.31E-1	4.57E-6	1.91E-10	2.11E-14	1.22E-2	**0**
F12	AVG	9.53E-1	9.95E-1	2.45	5.82E-2	9.30E-1	**6.84E-11**	7.23	2.07E-5
	STD	1.89E-1	1.65E-1	9.21E-1	1.38E-2	2.00E-1	**2.29E-11**	2.94	6.02E-6
F13	AVG	2.65	2.83	7.12	7.28E-2	2.88	1.02E-2	7.55	**4.71E-5**
	STD	3.47E-1	8.12E-2	4.67	8.48E-4	2.06E-1	6.17E-3	1.10E-2	**3.18E-5**
F14	AVG	**1.00**	**1.00**	**1.00**	**1.00**	**1.00**	**1.00**	**1.00**	**1.00**
	STD	**0**	**0**	9.95E-5	**0**	6.20E-6	**0**	2.09E-6	**0**
F15	AVG	**1.35E-31**	1.48E-1	**1.35E-31**	9.35E-2	2.24E-1	1.59E-13	1.55E-13	7.78E-5
	STD	**6.57E-47**	9.75E-2	**6.57E-47**	8.94E-2	2.45E-1	1.37E-13	1.40E-13	4.63E-5
F16	AVG	**− 1.03**	− 1.03	**− 1.03**	**− 1.03**	− 7.81E-1	**− 1.03**	**− 1.03**	− 8.54E-1
	STD	**4.44E-16**	4.07E-3	**4.44E-16**	5.21E-3	3.03E-1	**4.44E-16**	**4.44E-16**	2.84E-1
F17	AVG	**3.98E-1**	5.02E-1	**3.98E-1**	4.92E-1	4.43E-1	**3.98E-1**	**3.98E-1**	7.14E-1
	STD	**5.55E-17**	1.07E-1	**5.55E-17**	1.18E-1	1.02E-1	**5.55E-17**	**5.55E-17**	1.56E-1
F18	AVG	3.90	3.28	**3.00**	3.15	4.08	**3.00**	**3.00**	2.22E + 1
	STD	4.85	9.53E-1	**0**	1.98E-1	2.64	**0**	**0**	1.21E + 1
F19	AVG	**− 3.86**	− 3.54	− 3.62	− 3.67	− 3.23	**− 3.86**	**− 3.86**	− 3.30
	STD	**1.78E-15**	2.89E-1	3.50E-1	2.45E-1	3.55E-1	**1.78E-15**	**1.78E-15**	3.98E-1
F20	AVG	**0**	**0**	**0**	**0**	2.27E-9	3.70E-18	2.82E-11	**0**
	STD	**0**	**0**	**0**	**0**	3.41E-9	1.38E-17	3.00E-11	**0**
F21	AVG	**1.00**	**1.00**	1.38E + 8	**1.00**	**1.00**	**1.00**	3.83E + 5	**1.00**
	STD	**0**	**0**	9.05E + 7	**0**	**0**	**0**	4.30E + 5	**0**
F22	AVG	4.95	5.00	2.80E + 4	4.06E + 2	5.00	**4.80**	8.49E + 2	5.00
	STD	1.30E-1	5.30E-3	8.08E + 3	2.98E + 2	7.47E-5	2.79E-1	5.09E + 2	**0**
F23	AVG	**1.98**	9.34	1.23E + 1	4.57	7.68	9.16	4.15	1.17E + 1
	STD	1.08	7.50E-1	**6.25E-1**	2.81	1.62	8.20E-1	2.04	1.16
F24	AVG	**1.19E + 1**	1.02E + 2	1.03E + 2	1.83E + 1	1.09E + 2	3.38E + 1	2.77E + 1	1.34E + 2
	STD	**4.81**	1.47E + 1	1.00E + 1	6.31	2.29E + 1	1.33E + 1	1.11E + 1	1.93E + 1
F25	AVG	**1.01**	7.36E + 1	8.02E + 1	1.13	8.39E + 1	1.17	1.21	1.52E + 2
	STD	**9.12E-3**	2.50E + 1	2.53E + 1	7.92E-2	3.49E + 1	9.94E-2	9.66E-2	3.29E + 1
F26	AVG	**1.05**	1.16E + 1	1.12E + 1	3.58	1.20E + 1	5.44	3.94	1.36E + 1
	STD	**2.69E-1**	8.76E-1	8.66E-1	1.22	1.12	1.67	1.52	1.02
F27	AVG	2.12E + 3	2.18E + 3	1.08E + 3	**8.06E + 2**	2.05E + 3	1.04E + 3	9.70E + 2	2.72E + 3
	STD	**2.23E + 2**	2.30E + 2	3.74E + 2	3.15E + 2	4.29E + 2	2.91E + 2	3.04E + 2	2.65E + 2
F28	AVG	**3.97**	5.21	5.15	5.17	5.34	4.23	4.14	5.25
	STD	4.11E-1	1.45E-1	2.22E-1	1.36E-1	2.28E-1	3.10E-1	4.69E-1	**6.03E-2**
F29	AVG	**1.07**	3.76	3.49	1.22	5.06	1.25	1.29	4.81
	STD	**3.10E-2**	5.45E-1	7.32E-1	9.66E-2	6.12E-1	1.22E-1	1.20E-1	5.71E-1
F30	AVG	2.16E + 1	2.16E + 1	2.03E + 1	2.15E + 1	2.18E + 1	2.04E + 1	**1.91E + 1**	2.17E + 1
	STD	1.28E-1	1.23E-1	3.59	1.22E-1	8.59E-2	3.61	5.84	**5.19E-2**
	W/L/T	13/7/10	0/24/6	1/24/5	1/26/3	0/29/1	2/22/6	1/25/4	1/23/6
	OE	76.66%	20%	20%	13.33%	3.33%	26.66%	16.66%	**23.33%**

Significant values are in [bold].

**Table 5:** Result of different algorithms on F1-F13 with dimension 50.

		DGS-SCSO	SCSO	AEFA	HBA	HPSOBOA	QSSALEO	TBLSBCL	TSO
F1	AVG	**0**	1.06E-161	1.46E + 1	1.20E-61	2.88E-8	9.69E-14	4.38E-8	1.85E-134
	STD	**0**	**0**	2.10E-1	2.24E-62	1.24E-8	5.54E-14	8.36E-9	3.44E-135
F2	AVG	**6.19E-284**	3.78E-86	2.23E + 2	1.07E-34	3.71E + 23	2.23E-7	4.36	2.07E-68
	STD	**0**	7.02E-87	4.84E + 1	2.01E-35	1.65E + 23	2.22E-7	1.84	3.84E-69
F3	AVG	**0**	8.70E-126	4.87E + 3	1.38E-22	1.19E-6	1.21E-13	1.88E + 3	1.16E-137
	STD	**0**	1.61E-126	1.22E + 3	3.47E-23	4.72E-7	7.95E-14	6.02E + 2	2.15E-138
F4	AVG	**5.26E-268**	9.50E-71	8.02	5.92E-20	7.96E-6	1.64E-7	1.59E + 1	3.01E-66
	STD	**0**	1.77E-71	2.22	1.60E-20	5.52E-6	1.18E-7	2.55	6.41E-67
F5	AVG	**5.73E-273**	1.88E-167	6.69E + 2	6.72E-61	7.11E-7	7.39E-13	1.27E + 2	4.38E-137
	STD	**0**	**0**	6.23E + 2	1.25E-61	4.60E-7	6.63E-13	9.73E + 1	8.14E-138
F6	AVG	1.12E + 1	1.11E + 1	1.36E + 1	9.65E-2	1.14E + 1	5.29E-8	**4.30E-8**	7.02E-5
	STD	6.34E-1	4.25E-1	1.02E + 1	7.50E-3	5.66E-1	1.33E-8	**6.93E-9**	4.10E-5
F7	AVG	**2.04E-5**	4.31E-5	4.21E + 2	4.06E-3	4.49E-4	1.07E-4	2.30E-1	7.58E-5
	STD	**1.82E-5**	4.18E-5	6.76E + 1	5.80E-4	3.56E-4	9.56E-5	6.24E-2	6.03E-5
F8	AVG	**0**	8.97E-76	3.15	9.63E-2	7.33E-6	3.62E-8	2.21	1.44E-64
	STD	**0**	1.67E-76	5.08E-1	8.84E-3	6.35E-6	2.82E-8	3.31E-1	2.67E-65
F9	AVG	**0**	**0**	1.82E + 2	1.26E	9.64E-6	3.41E-14	7.56E + 1	**0**
	STD	**0**	**0**	4.54E + 1	2.57E-1	5.46E-6	1.14E-14	1.37E + 1	**0**
F10	AVG	**4.44E-16**	**4.44E-16**	2.86	9.03	3.17E-5	7.48E-8	3.89	**4.44E-16**
	STD	**9.86E-32**	**9.86E-32**	1.10	8.67	3.14E-5	5.96E-8	8.42E-1	**9.86E-32**
F11	AVG	**0**	**0**	8.29	7.97E-17	3.57E-10	6.20E-14	8.34E-3	**0**
	STD	**0**	**0**	4.10	1.48E-17	1.88E-10	4.01E-14	1.10E-4	**0**
F12	AVG	9.93E-1	1.13	6.82	5.62E-3	1.09	**2.15E-10**	1.05E + 1	3.51E-6
	STD	1.58E-1	1.01E-1	3.58	1.17E-4	1.27E-1	**7.30E-11**	3.34	7.10E-6
F13	AVG	4.69	4.85	7.30E + 1	1.26	4.91	5.08E-2	6.25E + 1	**6.20E-5**
	STD	3.88E-1	4.51E-2	3.95E + 1	5.67E-1	1.79E-1	4.39E-2	2.18E + 1	**4.77E-5**
	W/L/T	7/3/3	0/13/3	0/13/0	0/13/0	0/13/0	1/12/0	1/12/0	1/9/3
	OE	76.92%	0%	0%	0%	0%	7.69%	7.69%	30.76%

Significant values are in [bold].

**Table 6:** Result of different algorithms on F1-F13 with dimension 100.

		DGS-SCSO	SCSO	AEFA	HBA	PSOBOA	QSSALEO	TBLSBCL	TSO
F1	AVG	**0**	7.68E-141	1.01E + 3	1.55E-62	1.25E-8	1.99E-13	2.27E-1	4.87E-119
	STD	**0**	1.43E-141	3.18E + 2	4.70E-63	1.05E-8	1.09E-13	1.72E-1	9.04E-120
F2	AVG	**6.24E-261**	2.92E-71	4.41E + 2	1.38E-33	7.38E + 48	4.18E-7	2.02E + 1	7.30E-64
	STD	**0**	5.42E-72	4.06E + 1	2.64E-34	1.63E + 48	3.43E-7	1.04E + 1	1.36E-64
F3	AVG	**0**	7.09E-130	1.74E + 4	1.31E-3	2.42E-6	1.91E-12	1.42E + 4	1.69E-116
	STD	**0**	1.32E-130	4.89E + 3	2.44E-4	1.17E-6	9.08E-13	2.76E + 3	3.14E-117
F4	AVG	**7.23E-227**	2.98E-66	1.67E + 1	4.01E-16	7.81E-6	2.09E-7	1.85	1.85E-73
	STD	**0**	5.54E-67	2.07	8.24E-17	6.12E-6	1.48E-7	2.62E + 1	3.45E-74
F5	AVG	**1.98E-240**	2.33E-158	2.69E + 4	2.70E-55	8.23E-6	7.56E-12	3.95E + 3	1.42E-131
	STD	**0**	**0**	6.50E + 3	5.04E-56	5.90E-6	3.84E-12	1.88E + 3	2.66E-132
F6	AVG	2.31E + 1	2.36E + 1	1.01E + 3	4.21	2.40E + 1	**6.89E-7**	2.47E-1	7.74E-4
	STD	1.20	3.99E-1	3.18E + 2	9.29E-1	4.71E-1	**1.55E-7**	2.39E-1	2.34E-4
F7	AVG	**1.80E-5**	3.83E-5	1.91E + 3	7.48E-3	4.98E-4	1.00E-4	1.14	6.76E-5
	STD	**1.71E-5**	3.51E-5	1.25E + 2	4.63E-3	7.39E-4	8.55E-5	2.29E-1	6.09E-5
F8	AVG	**4.16E-130**	1.23E-80	7.43	9.09E-2	7.70E-6	6.23E-8	7.14	1.11E-66
	STD	**7.73E-131**	2.30E-81	7.92E-1	4.81E-2	7.33E-6	4.42E-8	7.42E-1	2.06E-67
F9	AVG	**0**	**0**	7.97E + 2	2.80E-1	3.37E-5	9.50E-14	1.52E + 2	**0**
	STD	**0**	**0**	1.01E + 2	7.46E-2	1.56E-5	4.17E-14	2.69E + 1	**0**
F10	AVG	**4.44E-16**	**4.44E-16**	7.98	1.46E + 1	2.28E-5	8.75E-8	7.55	**4.44E-16**
	STD	**9.86E-32**	**9.86E-32**	7.66E-1	7.61	1.97E-5	5.12E-8	1.54	**9.86E-32**
F11	AVG	**0**	**0**	4.84E + 1	**0**	2.78E-10	2.20E-13	1.95E-1	**0**
	STD	**0**	**0**	1.09E + 1	**0**	1.54E-10	1.06E-13	6.28E-2	**0**
F12	AVG	1.09	1.16	3.12E + 2	5.27E-2	1.11	**2.69E-8**	1.67E + 1	2.95E-6
	STD	1.33E-1	6.93E-2	1.54E + 2	1.35E-2	8.33E-2	**3.62E-8**	3.60	8.86E-7
F13	AVG	9.74	9.87	3.83E + 5	7.16	9.95	5.44E-1	1.73E + 2	**4.96E-5**
	STD	2.77E-1	4.78E-2	3.13E + 5	1.35	1.53E-1	2.40E-1	2.07E + 1	**3.71E-5**
	W/L/T	7/3/3	0/10/3	0/13/0	0/12/1	0/13/0	2/11/0	0/13/0	1/9/3
	OE	76.92%	23.07%	0%	7.69%	0%	15%	0%	30.76%

Significant values are in [bold].

**Figure 4:**
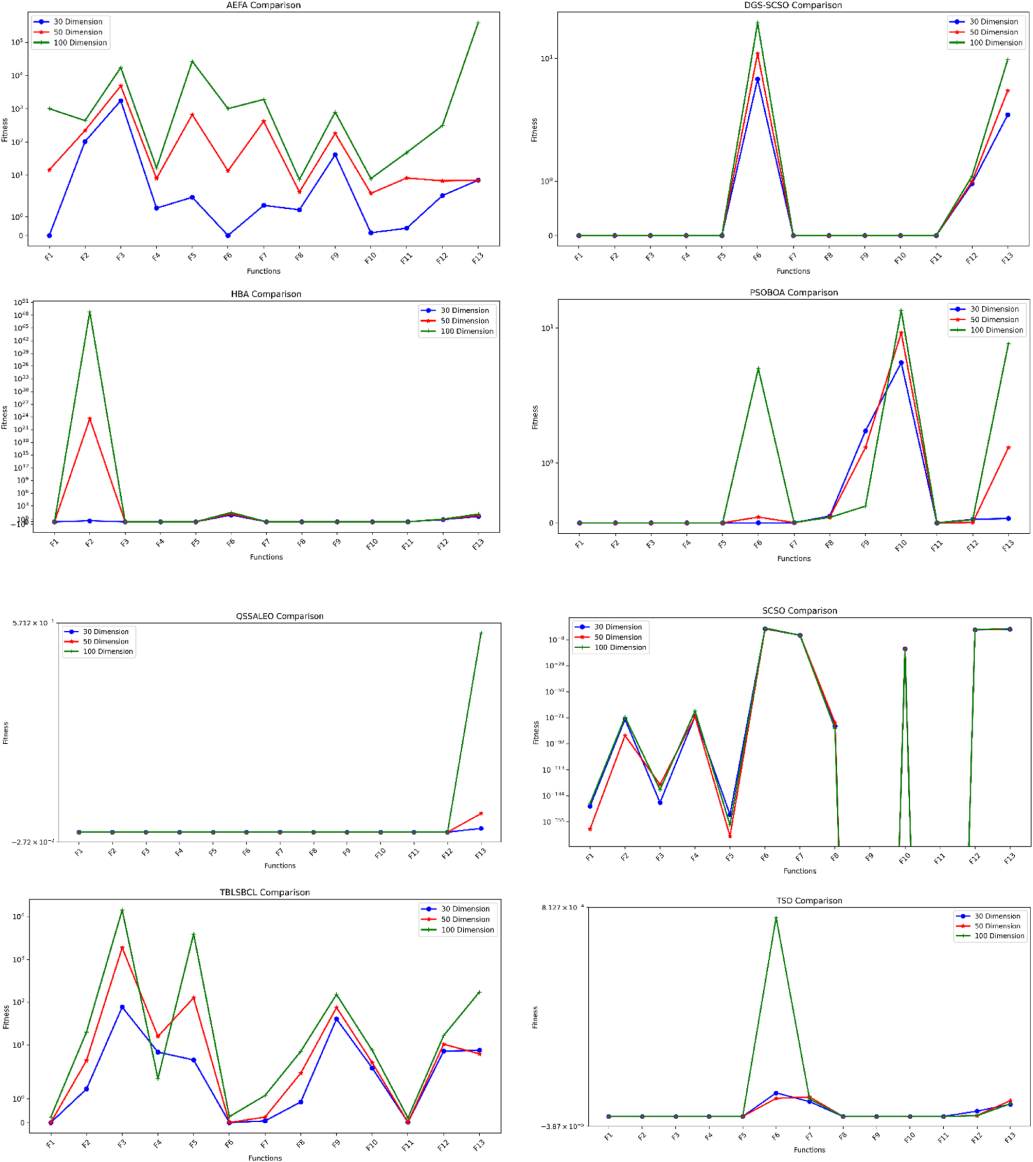
Result of different algorithms on 30 functions.

Furthermore, Table 4 displays the outcomes of various algorithms in tackling the 30-function test suite. It is clear that the improved DGS-SCSO algorithm has the best performance, achieving an overall efficiency (OE) of 79.66%, which considers the number of losses (L) and the total number of functions (NF). L is subtracted from NF, and the result of the subtraction is divided by NF to compute OE^42,45^. The table presents the OE of all the optimizers, denoting the number of “Wins, Losses, and Ties” as W, L, and T, respectively. In contrast to the traditional SCSO algorithm, which has an overall efficiency of 20% for all functions, DGS-SCSO has improved OE with a margin of 59.66% over SCSO. The integration of the both methods into SCSO algorithm has significantly improved the solution precision, resulting in better exploitation for unimodal functions, better exploration for multimodal functions, and a better tradeoff between the two in complex CEC 2019 functions.Additionally, in Tables 5 and 6, with increased dimension, DGS-SCSO maintains competitive AVG, low STD, and high overall efficiency (OE) across functions F1-F13 at dimensions 50 and 100, respectively, outperforming or performing comparably to other algorithms such as HBA, HPSOBOA, QSSALEO, TBLSBCL, and TSO in functions F1-F5, F7 and F8. The algorithm's ability to consistently achieve low AVG, low STD, and strong OE underscores its effectiveness, scalability, and reliability in addressing optimization challenges across diverse scenarios and dimensionalities. The results of the performance of each of the compared algorithms and DGS-SCSO on the scaled functions (F1-F13) from Tables 4, 5, and 6 are illustrated in Fig. 4 to visualize the consistency of each optimizer as complexity increases. As seen from the illustration, DGS-SCSO, QSSALEO, HBA, and TBLSBCL show relative consistency in their performance as the dimension increases; this demonstrates the robustness of DGS-SCSO.

### Nonparametric test analysis

The Wilcoxon Rank Test (WRT) is useful for analyzing data with complex distributions. Tables 4, 5, and 6 offer statistics on the average value and standard deviation of all the optimizers, but they do not allow for comparison between multiple algorithms. To verify and test the results, it is necessary to use the WRT. In Table 7, the outcomes of the DGS-SCSO algorithm and seven other algorithms are presented. These algorithms were run thirty times using thirty different benchmark functions with varying dimensions. A significance level (*P*-value) of 5% is used, and outcomes below this value indicate a "significant difference" among the two algorithms. Table 7 shows that most test results are below 5%, but few are above, meaning no significant difference. The QSSALEO and TSO algorithms have few results that are better than DGS-SCSO, as indicated by the "-" column. This suggests that these algorithms have good convergence on certain functions, which confirms the No-free-Lunch theorem, "stating that no single optimization algorithm can be applied to solve all types of optimization problems". However, it is worth noting that in the “ + ” column, which denotes the better performance of DGS-SCSO in comparison to the other algorithms, DGS-SCSO consistently outperforms them. The “ = ” column indicates equal performance. Table 8 presents another nonparametric test called the Friedman Test, which ranks compared methods from least to highest. DGS-SCSO ranked first in all test scenarios with the highest list value in the Friedman rank.

**Table 7:** Wilcoxon rank test.

Dimension	DGS-SCSO vs	-	+	=	R-	R +	*p*-value
30	SCSO	1	21	8	15	238	2.95E-04
	AEFA	4	21	5	49	276	2.26E-03
	HPSOBOA	6	20	4	110	241	9.62E-02
	HBA	2	26	2	37	369	1.57E-04
	QSSALEO	7	18	5	134	191	4.43E-01
	TBLSBCL	4	22	4	66	285	5.42E-03
	TSO	3	21	6	48	252	3.57E-03
50	SCSO	1	9	3	8	47	4.69E-02
	AEFA	0	13	0	0	91	1.47E-03
	HPSOBOA	3	10	0	33	58	3.82E-01
	HBA	0	13	0	0	91	1.47E-03
	QSSALEO	3	10	0	36	55	5.07E-01
	TBLSBCL	1	12	0	8	83	8.78E-03
	TSO	3	7	3	27	28	9.59E-01
100	SCSO	0	10	3	0	55	5.06E-03
	AEFA	0	13	0	0	91	1.47E-03
	HPSOBOA	3	9	1	31	47	5.30E-01
	HBA	0	12	0	0	78	2.22E-03
	QSSALEO	3	10	0	36	55	5.07E-01
	TBLSBCL	1	12	0	9	82	1.07E-02
	TSO	3	7	3	27	28	9.59E-01

**Table 8:** Friedman test.

		DGSSCSO	SCSO	AEFA	HPSOBOA	HBA	QSSALEO	TBLSCL	TSO
30dim	Friedman value	2.47	4.37	5.53	4.15	6.1	3.63	5.02	4.73
	Friedman rank	1	4	7	3	8	2	6	5
50dim	Friedman value	2.08	2.85	7.62	4.62	5.85	3.92	6.62	2.46
	Friedman rank	1	3	8	5	6	4	7	2
100dim	Friedman value	2.13	3.04	7.92	4.71	5.58	3.67	6.58	2.38
	Friedman rank	1	3	8	5	6	4	7	2

### Convergence curve analysis

**Figure 5:**
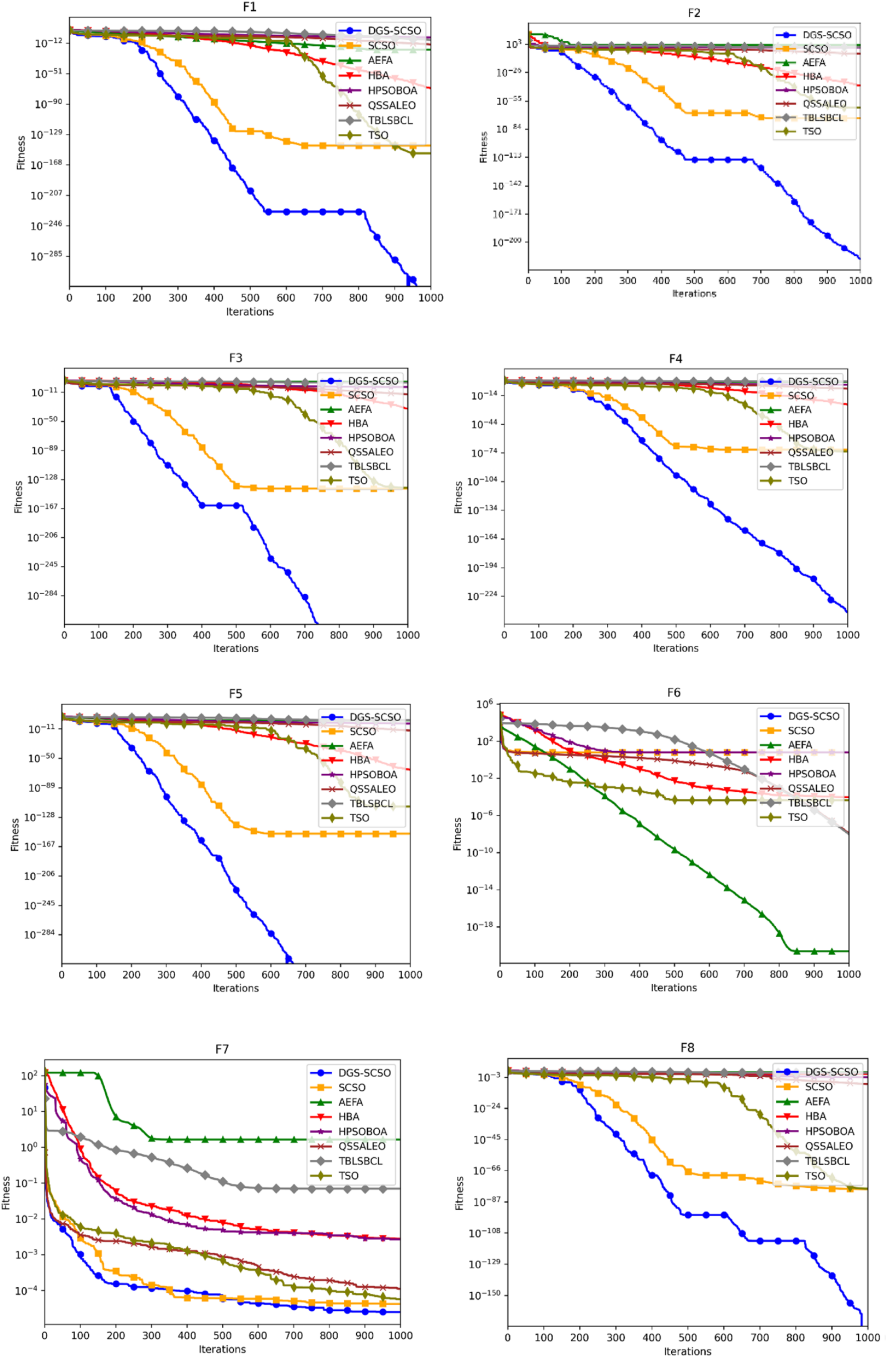

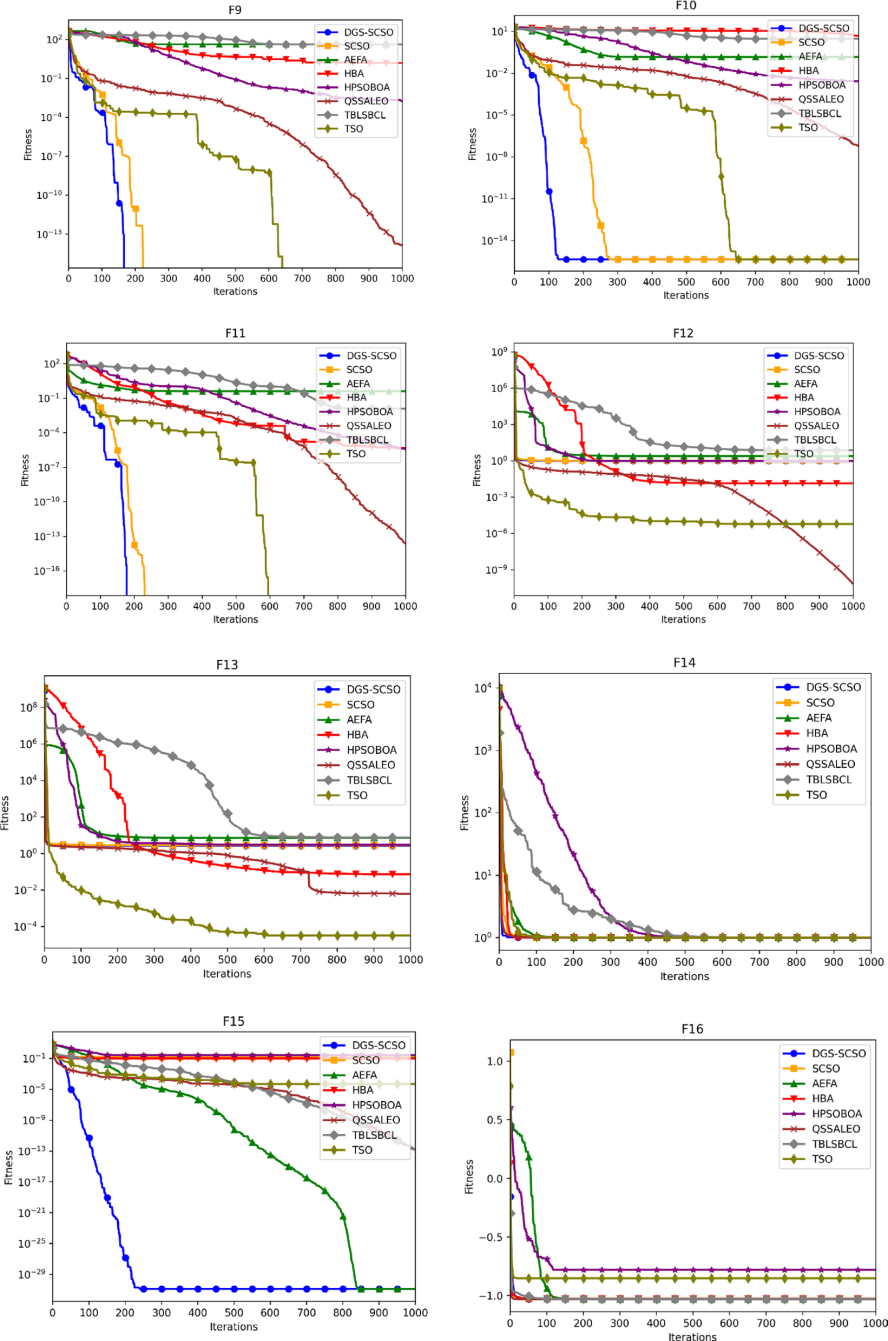

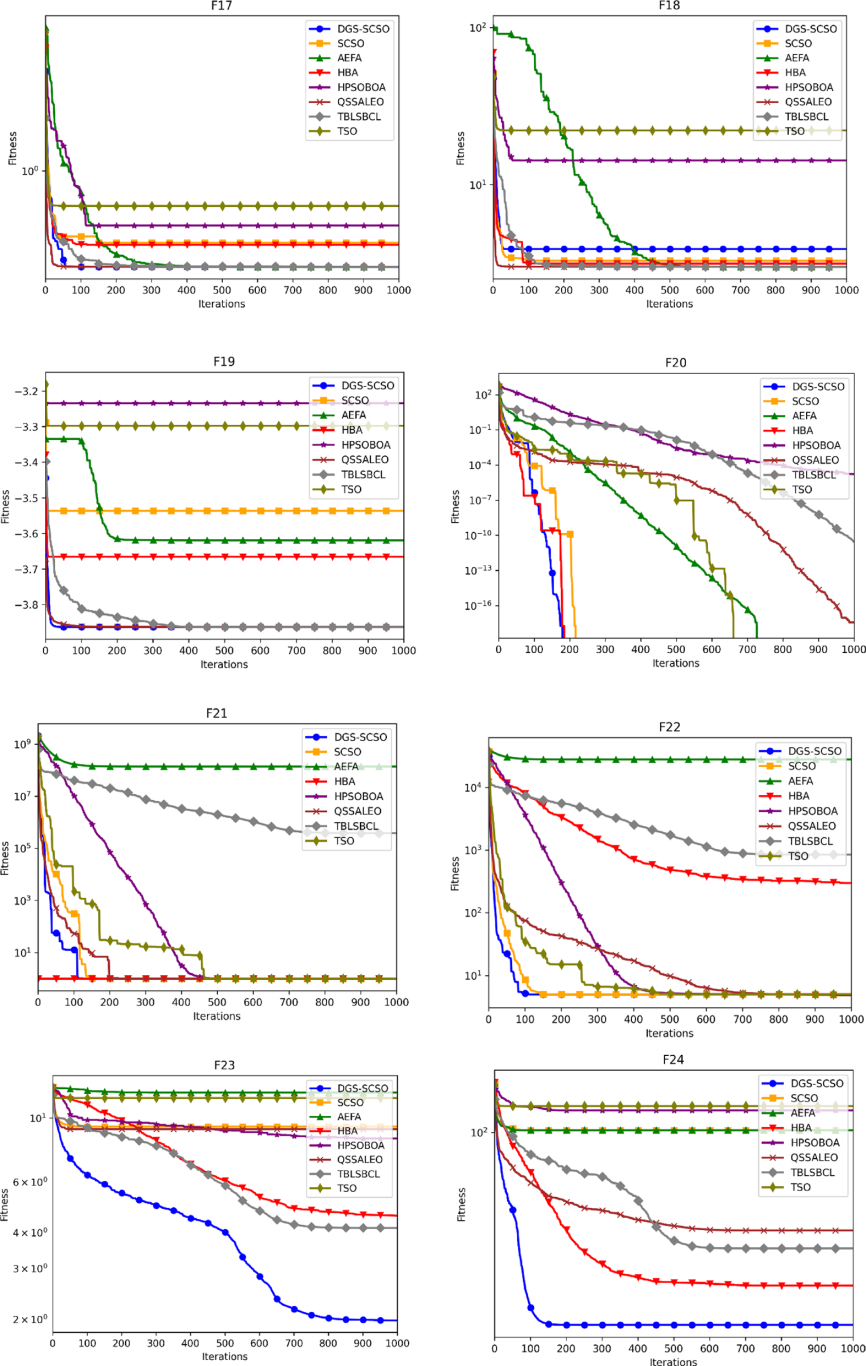

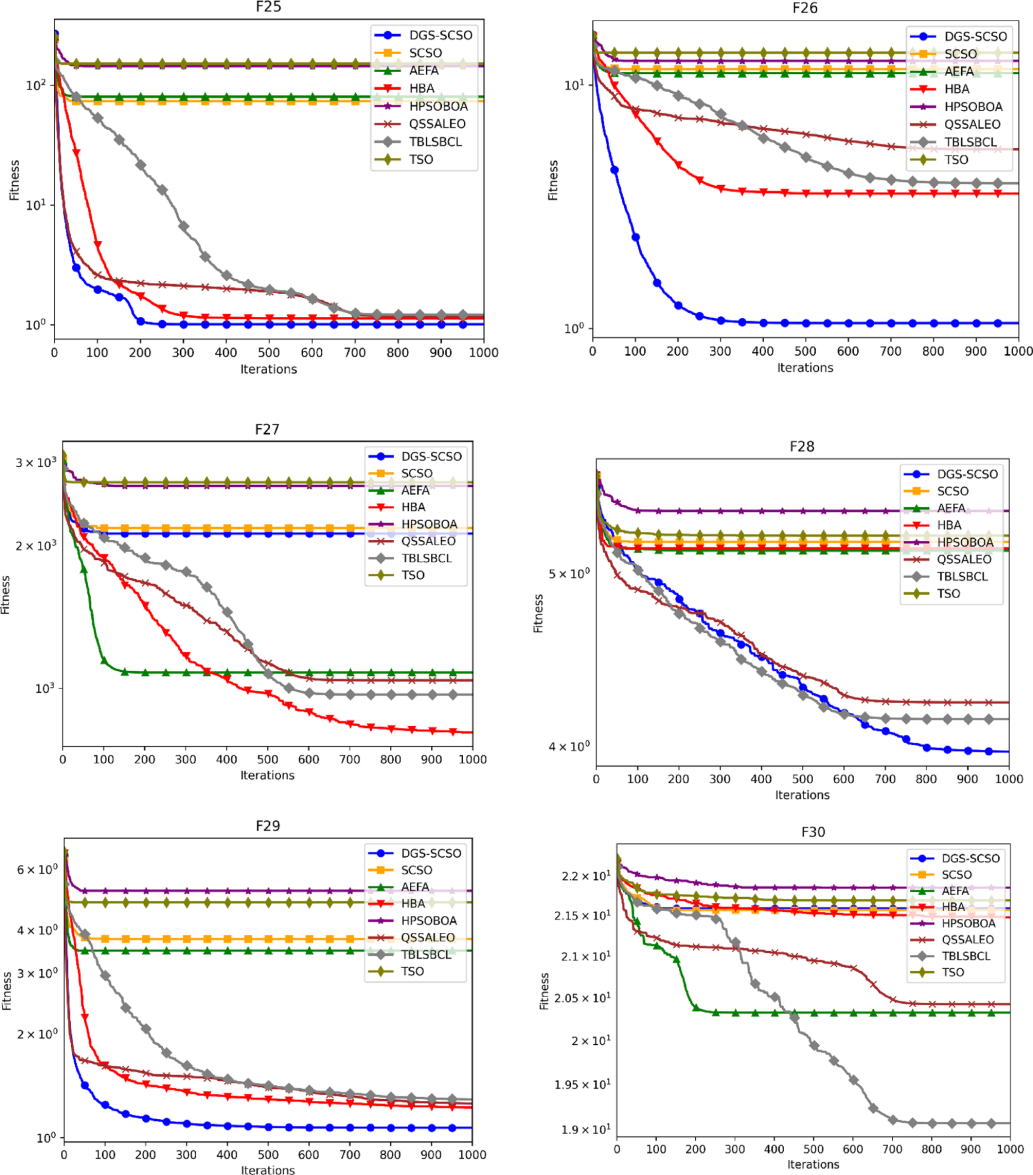
Convergence curve for functions F1 to F30.

Figure 5 depicts the average convergence profiles of various optimization algorithms across 30 independent runs using the dimensions of Tables 1 and 2. The efficacy and efficiency of an optimization algorithm can be assessed using the speed and accuracy of its convergence towards the optimal solution, as reflected in its convergence trajectory. In this regard, the DGS-SCSO algorithm performs better than the original SCSO algorithm, achieving faster convergence rates, particularly in the initial search phases. The proposed algorithm demonstrates notable improvement in convergence performance for most functions, indicating its effectiveness in enhancing the optimization process. Specifically, in unimodal functions F1-F5 and F7, the DGS-SCSO algorithm converges far more rapidly than other algorithms in the initial iterations, achieving the best convergence precision compared to other algorithms. On multimodal functions, the DGS-SCSO algorithm maintains superior convergence speed and accuracy across most functions. Notably, in F8-10, F15, and F20, the algorithm performs exceptionally well, reaching the proximity of the global optimum and surpassing other optimizers. The incorporation of Gold-SA techniques enables the algorithm to rapidly track the best solution to speed up the convergence in the initial search stages for unimodal functions. The DPI method facilitates the algorithm's breakout from the local optimum in multimodal functions, contributing to its outstanding performance. In terms of convergence accuracy on complex functions, the DGS-SCSO algorithm outperforms other algorithms. Specifically, in F23-F29, DGS-SCSO demonstrates superior performance compared to other novel optimization algorithms.

### Box plot analysis

**Figure 6:**
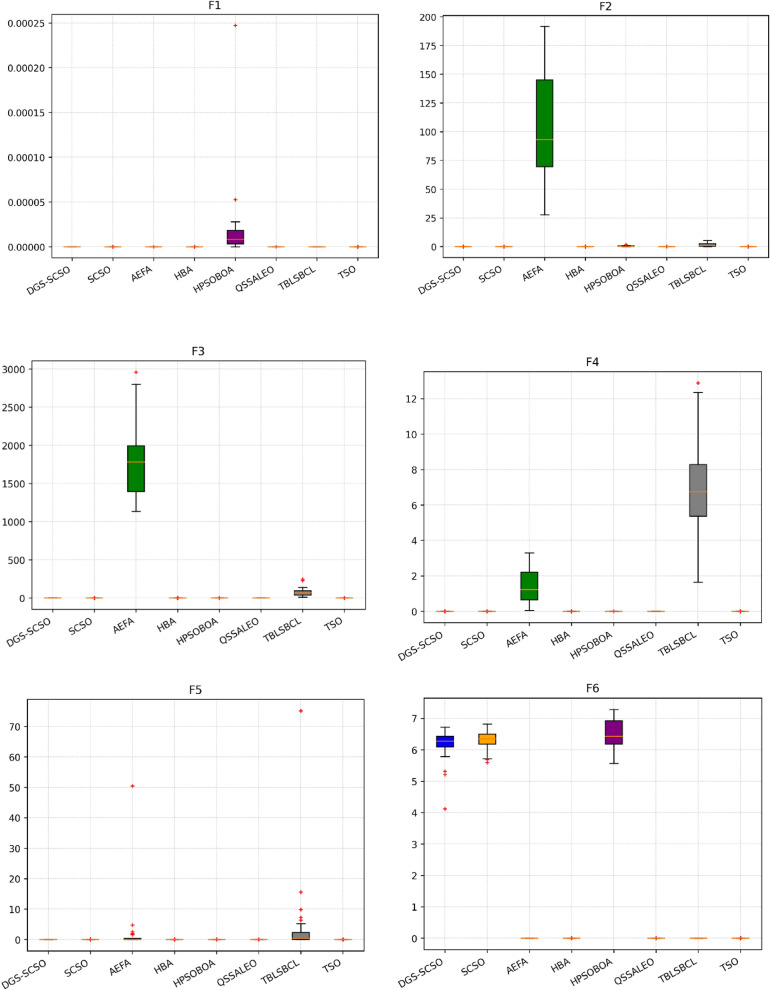

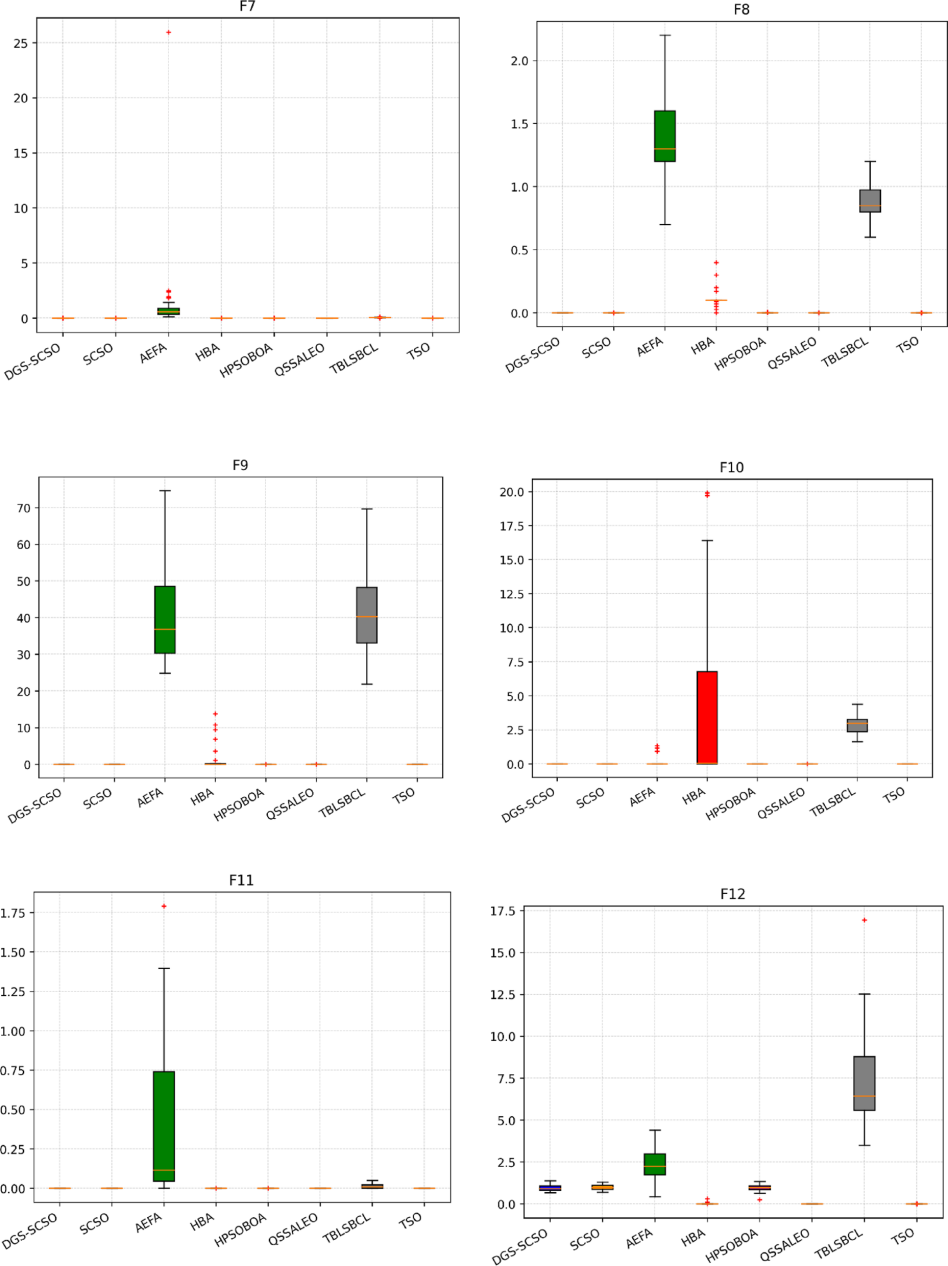

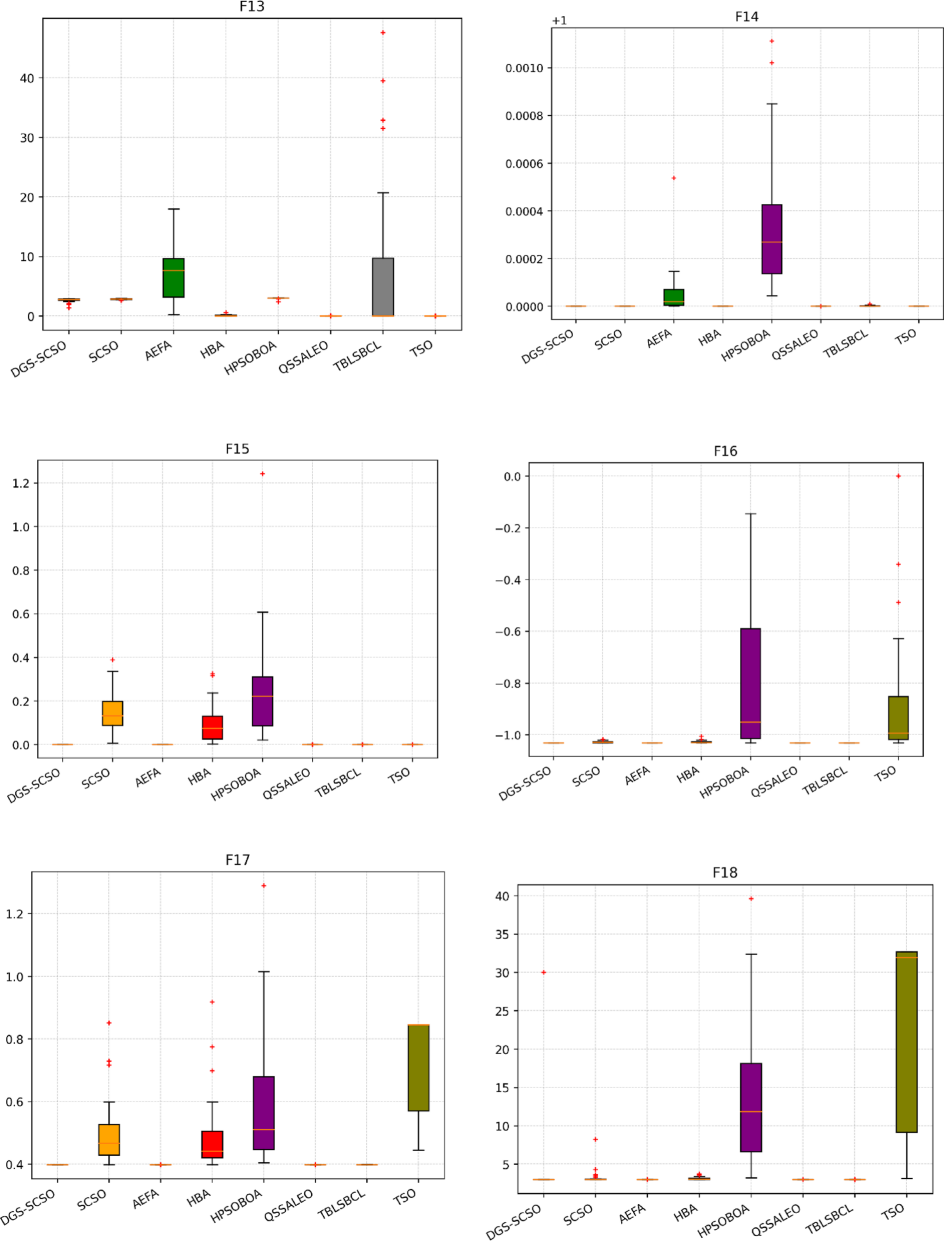

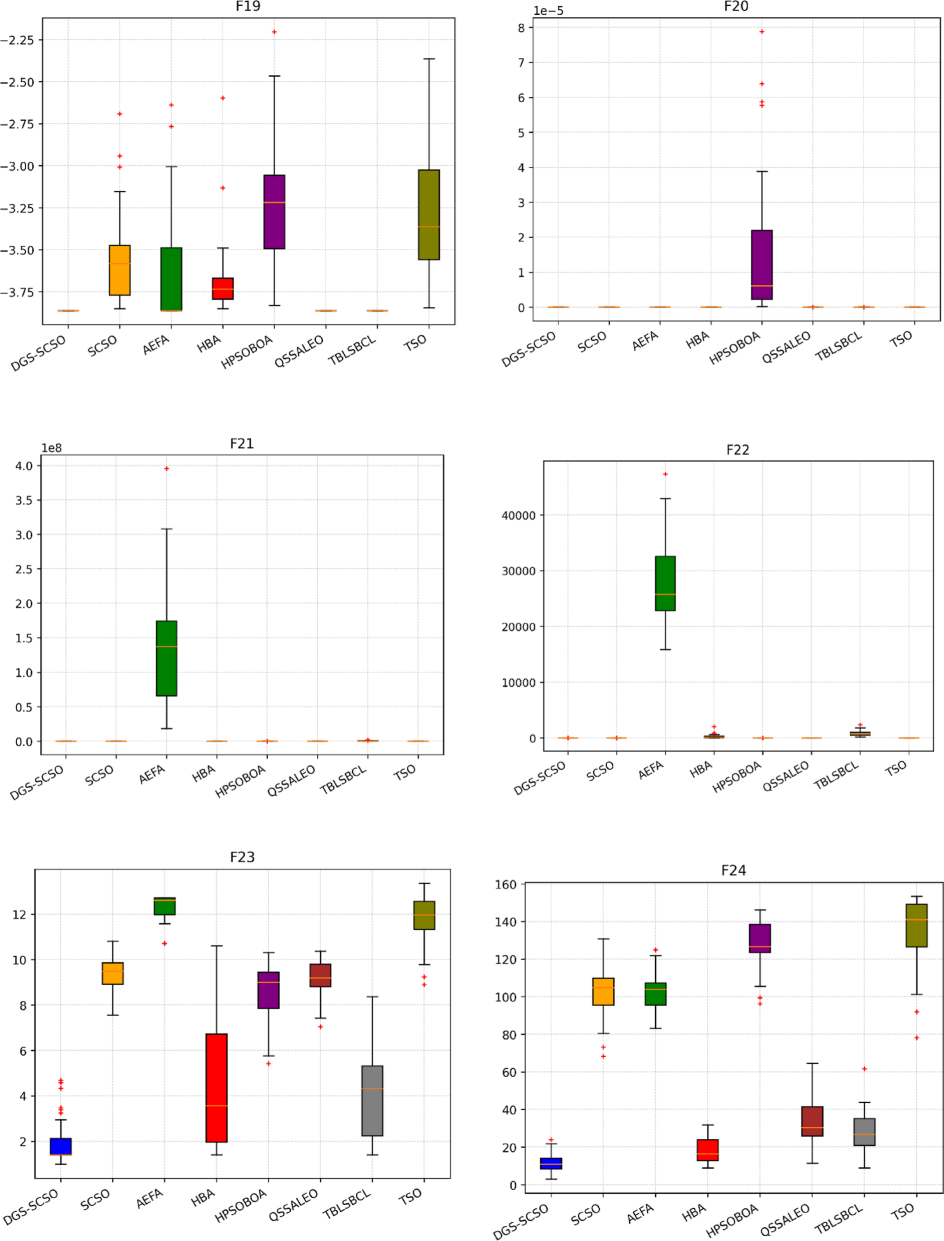

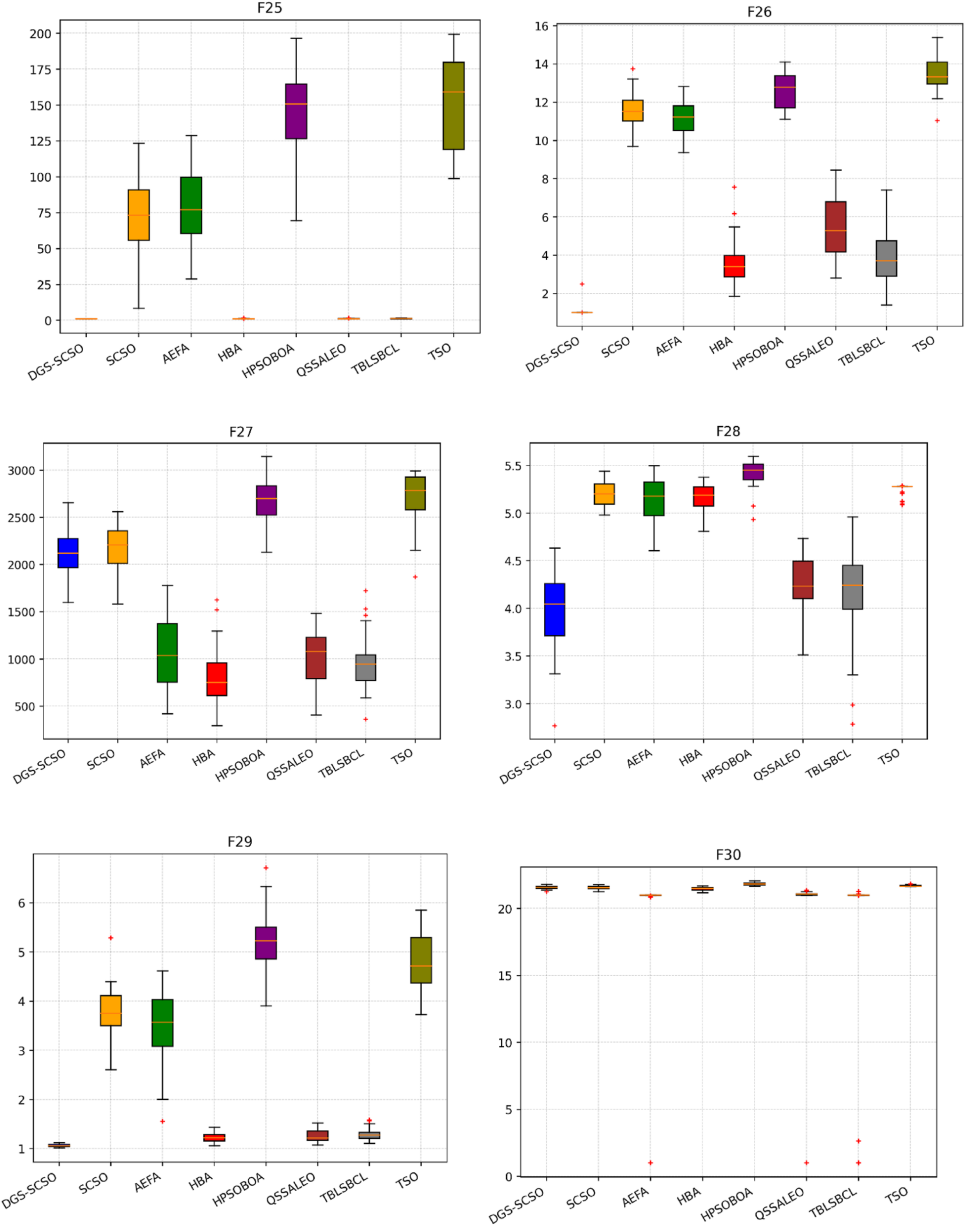
Boxplot plots on benchmark functions F1 to F30.

Through boxplot analysis, the distributional properties of the data may be shown. The data distribution is shown as quartiles in the boxplot. The algorithm's lowest and highest values are found at the lowest and highest points of the boxplot. The rectangle's ends serve as a boundary between the lower and upper quartiles. In this section of the study, the boxplot behaviour was used to demonstrate the distribution of the obtained value for each algorithm. The benchmark functions were run independently 30 times for each sample using the dimensions in Tables 1 and 2. From Fig. 6, it can be concluded that DGS-SCSO demonstrated better stability for most benchmark functions and outperformed the other algorithms. This indicates that DGS-SCSO is a more reliable and consistent algorithm for finding the global optimum. The boxplot for the proposed DGS-SCSO method was narrow in most cases for F1 to F20 and comparable to other algorithms. This indicates that the DGS-AEFA method performs well for less complicated functions and maintains performance where the global optimum is easier to find. DGS-SCSO had lower values in much more complex functions like F23, F24, F26, F28, and F29 than all other algorithms. This suggests that DGS-SCSO is also able to maintain stability and is able to handle more complex functions well where finding the global optimum is more challenging. Overall, DGS-SCSO shows an advantage in stability and robustness when taking into account the “length and median” of the box, which is the thin line inside the box. The addition of the two enhancement techniques led to greater harmony between the exploitation and exploration capacities, making the algorithm more efficient as a whole.

### Exploration and exploitation analysis

Too much exploration can lead to inefficient search and slow convergence, while too much exploitation can result in early convergence to local optima and a failure to discover better solutions. In this subsection, we observe the exploitation and exploration capability of the proposed method as proposed by Kashif et al^46^.

13 $${Div}_{j}=\frac{1}{n}\sum_{i=1}^{n} {\text{median}}\left({x}^{j}\right)-{x}_{i}^{j}$$

14 $$Div=\frac{1}{D}\sum_{j=1}^{D} {Div}_{j}$$

In Eq. (13), the population’s diversity in dimension $j$ is measured. To compute the diversity of a single dimension $j$, firstly we find the median value denoted as ${\text{median}}\left({x}^{j}\right)$ of that dimension across all individuals $n$ in the swarm. Subsequently, we compute the distance of every individual $i$ value for that dimension from the median value $j$, and take the average of these distances across all individuals ${{\text{Div}}}_{j}$ in the swarm in Eq. (14). This gives diversity ${{\text{Div}}}_{j}$ for that dimension. To compute the overall diversity $Div$ of the swarm, we repeat this process for each dimension $j$, and then take the average of the diversities ${{\text{Div}}}_{j}$ across all dimensions. The purpose of this calculation is to measure how diverse the individuals in the swarm are in terms of their dimensional values. If all individuals have very similar values for all dimensions, then the diversity will be low. If there is a lot of variation in the values across dimensions and individuals, then the diversity will be high. Equations (14) and (15) determine the exploration and exploitation percentages in an iteration:

15 $$\mathrm{Exploration\%}=\frac{Div}{Di{v}_{max}}\times 100$$

16 $$\mathrm{Exploitation\%}=\frac{\mid \text{ Div }-{\text{ Div }}_{max}\mid }{{\text{ Div }}_{max}}\times 100$$

where, $Div$ is the diversity of the swarm in the current iteration, $Di{v}_{max}$ is the maximum diversity among all iterations, $\mathrm{Exploration\%}$ is the percentage of exploration in the current iteration, and $\mathrm{Exploitation\%}$ is the percentage of exploitation in the current iteration.

**Figure 7:**
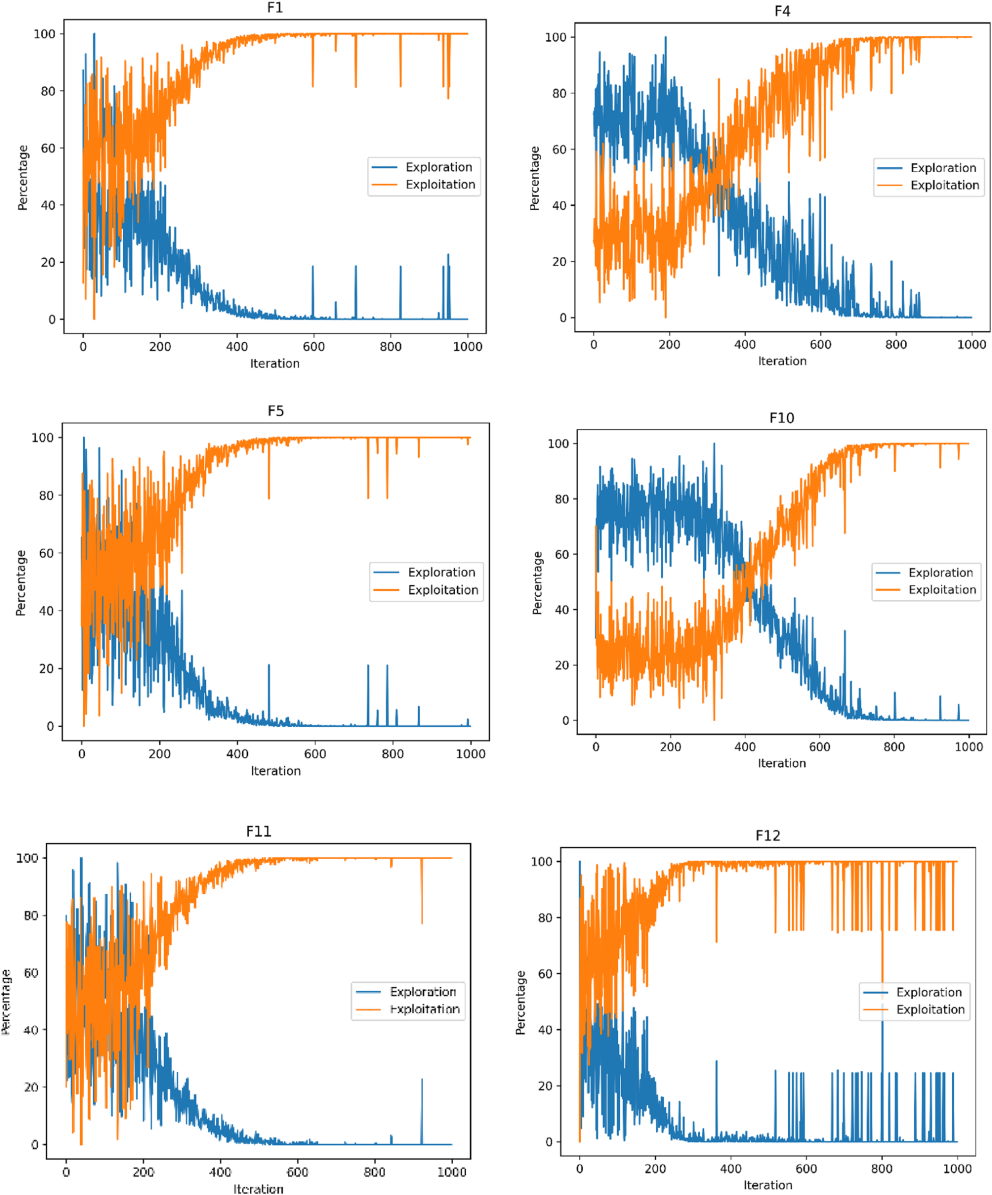
Exploitation and exploration plot of DGS-SCSO.

In Fig. 7, we used unimodal functions F1, F4, and F5 to depict how well the optimizer is able to explore. On the other hand, the multimodal functions F10, F11, and F12 in Fig. 6 depict how well the optimizer is capable to explore the search area. It can be observed that the method begins with a wide exploration and narrow exploitation of the functions examined. The appropriate balance between both optimization processes is seen as the iteration process progresses.

## Application of engineering problem

In this section, DGS-SCSO is compared to seven other algorithms on popular engineering problems, the experiment settings are the same as the previous experiment.

### Tension/compression spring design problem (TCSD)

**Figure 8:**
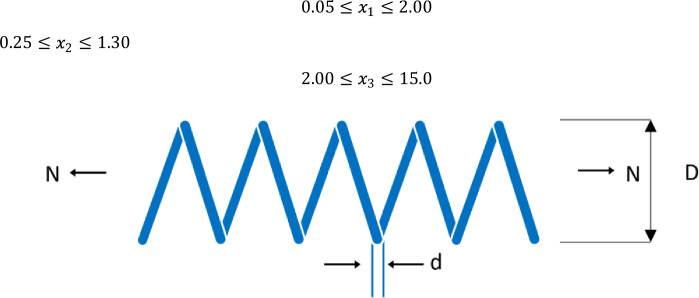
Tension/compression spring design problem parameters.

The TCSD problem evaluated in this subsection is a continuous constrained problem that minimizes the weight of a TCSD, as illustrated in Fig. 8. It includes three parameters: the number of “active coils (N), the mean coil diameter (D), and the wire diameter (d)”; with three constraining factors: "minimum deflection, shear stress, and urge frequency". We further proceeded to apply the DGS-SCSO algorithm and other metaheuristic algorithms to solve the TCSD problem. The results provided in Table 9 show that the DGS-SCSO algorithm outperformed the other algorithms in determining the optimum cost^47^.

**Table 9:** Results of tension/compression spring design problem.

Optimizers	Optimal Cost	d	D	N
DGS-SCSO	**0.012665233**	0.051683015	0.356572307	11.29749715
SCSO	0.01287564	0.052802393	0.381809003	10.09526686
AEFA	0.012704376	0.050244994	0.322960141	13.58184231
HBA	0.012814532	0.051761779	0.356086697	11.43162679
HPSOBOA	0.012745971	0.05	0.317013059	14.08258117
QSSALEO	0.013652141	0.058302711	0.531052593	5.562854585
TBLSBCL	0.012693336	0.050462415	0.327918248	13.20106881
TSO	0.012797224	0.05	0.316569921	14.1698549

Significant values are in [bold].

Considering the vector $\overrightarrow{x}=\left[{x}_{1}{x}_{2}{x}_{3}\right]=[dDN]$

We aim to minimize

18 $$f(\overrightarrow{x})=\left({x}_{3}+2\right){x}_{2}{x}_{1}^{2}$$

Constrained by

18 $${g}_{1}(\overrightarrow{x})=1-\frac{{x}_{2}^{3}{x}_{3}}{7178{x}_{1}^{4}}\le 0$$

19 $${g}_{2}(\overrightarrow{x})=\frac{4{x}_{2}^{2}-{x}_{1}{x}_{2}}{12566\left({x}_{2}{x}_{1}^{3}-{x}_{1}^{4}\right)}+\frac{1}{510{x}_{1}^{2}}-1\le 0$$

20 $${g}_{3}(\overrightarrow{x})=1-\frac{140.45{x}_{1}}{{x}_{2}^{2}{x}_{3}}\le 0$$

21 $${g}_{4}(\overrightarrow{x})=\frac{{x}_{1}+{x}_{2}}{1.5}-1\le 0$$

Possible boundaries of vector $\overrightarrow{x}$:

$$0.05\le {x}_{1}\le 2.00$$

$$0.25\le {x}_{2}\le 1.30$$

$$2.00\le {x}_{3}\le 15.0$$

### Three-bar truss design

**Figure 9:**
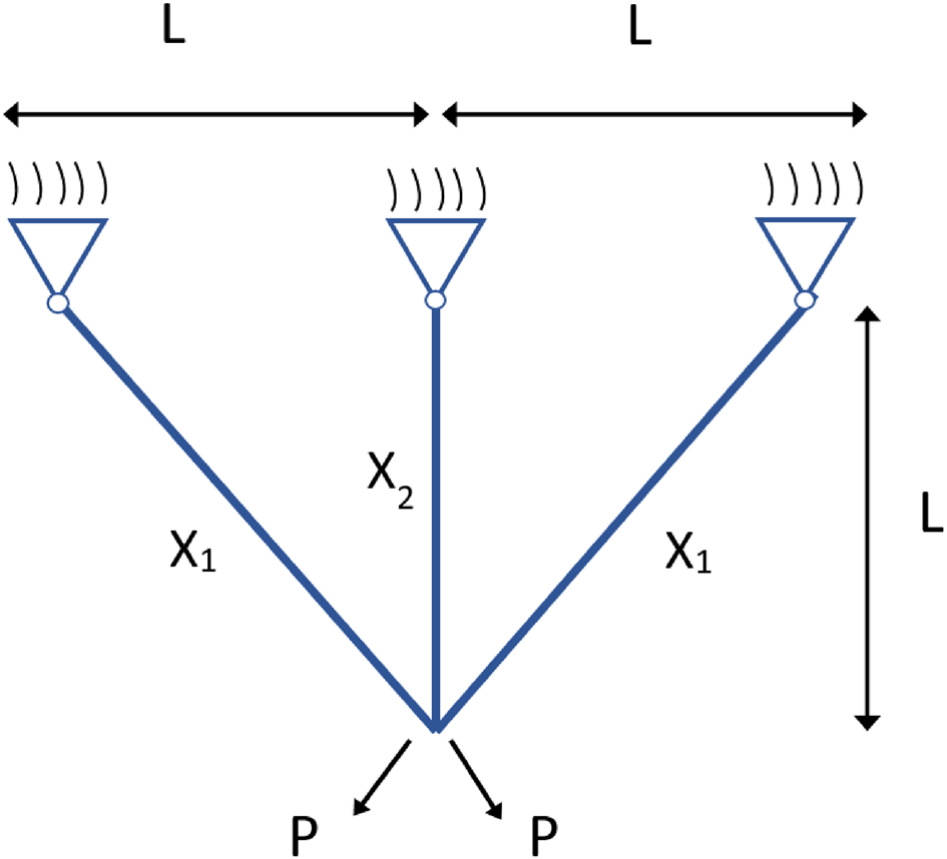
Three-bar truss design parameters.

Three bar truss design optimization problem's objective is to minimize the relevant weights related to the design illustrated in Fig. 9. The problem involves two optimization parameters (${x}_{1}$, ${x}_{2}$) and three constraining factors: buckling, deflection, and stress. The mathematical expression of the Three-bar truss design problem is presented below^48,49^:

22 $$Minimize:f({x}_{1},{x}_{2})=l\times \left(2\sqrt{2}{x}_{1}+{x}_{2}\right)$$

Constraining factors:

23 $${G}_{1}=\frac{\sqrt{2}{x}_{1}+{x}_{2}}{\sqrt{2}{x}_{1}2+2{x}_{1}{x}_{2}}P-\sigma \le 0$$

24 $${G}_{2}=\frac{{x}_{2}}{\sqrt{2}{x}_{1}2+2{x}_{1}{x}_{2}}P-\sigma \le 0$$

25 $${G}_{3}=\frac{1}{\sqrt{2}{x}_{2}+{x}_{1}}P-\sigma \le 0$$

where: $$l=100{\text{cm}};P=\frac{2kN}{{{\text{cm}}}^{2}};\sigma =\frac{2{\text{kN}}}{{{\text{cm}}}^{2}}$$

Interval: $$0\le {x}_{1},{x}_{2}\le 1$$

As seen in Table 10 DGS-SCSO obtained the best outcome for the optimal cost.

**Table 10:** Results of three bar truss design.

Optimizers	Optimal Cost	X_1_	X_2_
DGS-SCSO	**263.8958434**	0.788675135	0.408248288
SCSO	263.93549	0.790203018	0.404323249
AEFA	263.9700453	0.778799827	0.436921898
HBA	263.8976182	0.790203018	0.404323249
PSOBOA	263.8959836	0.789112361	0.407013031
QSSALEO	265.9728513	0.842073695	0.277984434
TBLSBCL	263.8961323	0.788050512	0.410017878
TSO	263.9017392	0.786501348	0.414455645

Significant values are in [bold].

## Conclusion

In conclusion, this paper introduces DGS-SCSO, a novel optimization algorithm that builds upon Sand Cat Swarm Optimization (SCSO) with the incorporation of Dynamic Pinhole Imaging (DPI) and Golden Sine Algorithm (Gold-SA). DPI improves global search capabilities and helps to avoid local optima, while Gold-SA addresses the drawbacks of SCSO, including early convergence and stagnation, thereby enhancing exploitation. The effectiveness of DGS-SCSO was assessed using 20 test functions and 10 CEC 2019 competition test functions, and the algorithm demonstrated superior optimization accuracy, convergence speed, robustness, and statistical significance when compared to other competitors. Furthermore, DGS-SCSO was evaluated on two real-world engineering design problems and significantly outperformed its peers. However, DGS-SCSO's time consumption is a potential concern due to its use of DPI and fitness evaluation to detect the best solutions, followed by the application of Gold-SA to improve the best solution. Future research will concentrate on reducing the computational time of DGS-SCSO while maintaining its performance, as well as exploring its applications to combinatorial optimization problems and coupling it with other optimizers to enhance its performance further. In addition to the aforementioned future directions, an online web server and an importable library will be developed to enhance the accessibility and usability of DGS-SCSO. Furthermore, our future efforts will focus on improving and advancing the constraint DGS-SCSO algorithm version, equipping it with enhanced techniques tailored for handling both equality and inequality constraints. These endeavours aim to strengthen the algorithm's applicability and performance across a broader range of real-world optimization problems.

## Data Availability

The datasets used and/or analyzed during the current study available from the corresponding author on reasonable request.
